# ERBB4 confers metastatic capacity in Ewing sarcoma

**DOI:** 10.1002/emmm.201202343

**Published:** 2013-05-16

**Authors:** Ariadna Mendoza-Naranjo, Amal El-Naggar, Daniel H Wai, Priti Mistry, Nikola Lazic, Fernanda Rocha Rojas Ayala, Isabela Werneck da Cunha, Pablo Rodriguez-Viciana, Hongwei Cheng, Jose H Tavares Guerreiro Fregnani, Patrick Reynolds, Robert J Arceci, Andrew Nicholson, Timothy J Triche, Fernando A Soares, Adrienne M Flanagan, Yuzhuo Z Wang, Sandra J Strauss, Poul H Sorensen

**Affiliations:** 1 UCL Cancer Institute, University College LondonLondon, UK; 2 Department of Molecular Oncology, BC Cancer Research CentreVancouver, BC, Canada; 3 Children's Hospital Los Angeles, Los AngelesCA, USA; 4 Department of Pathology, Hospital A.C CamargoSão Paulo, Brazil; 5 Texas Tech University, School of MedicineLubbock, TX, USA; 6 Kimmel Comprehensive Cancer Centre at Johns Hopkins, BaltimoreMD, USA; 7 Department of Histopathology, Royal Brompton HospitalLondon, UK

**Keywords:** ERBB4, Ewing sarcoma, FAK, metastasis, Rac1

## Abstract

Metastatic spread is the single-most powerful predictor of poor outcome in Ewing sarcoma (ES). Therefore targeting pathways that drive metastasis has tremendous potential to reduce the burden of disease in ES. We previously showed that activation of the ERBB4 tyrosine kinase suppresses *anoikis*, or detachment-induced cell death, and induces chemoresistance in ES cell lines *in vitro*. We now show that ERBB4 is transcriptionally overexpressed in ES cell lines derived from chemoresistant or metastatic ES tumours. ERBB4 activates the PI3K-Akt cascade and focal adhesion kinase (FAK), and both pathways contribute to ERBB4-mediated activation of the Rac1 GTPase *in vitro* and *in vivo*. ERBB4 augments tumour invasion and metastasis *in vivo*, and these effects are blocked by *ERBB4* knockdown. ERBB4 expression correlates significantly with reduced disease-free survival, and increased expression is observed in metastatic compared to primary patient-matched ES biopsies. Our findings identify a novel ERBB4-PI3K-Akt-FAK-Rac1 pathway associated with aggressive disease in ES. These results predict that therapeutic targeting of ERBB4, alone or in combination with cytotoxic agents, may suppress the metastatic phenotype in ES.

## INTRODUCTION

Ewing sarcoma (ES) is the second most common bone tumour in children and adolescents (Riggi & Stamenkovic, 2007). These tumours are characterized by etiologic gene fusions of *EWS* to different members of the *ETS* transcription factor family, and the expression of chimeric EWS–ETS fusion proteins such as EWS-FLI1 and EWS–ERG are pathognomonic of the disease (Toomey et al, 2010). Multi-modality therapy has greatly improved the outcome for ES patients, and those with localized disease at diagnosis have 5-year survival rates approaching 70% (Damron et al, 2007). However, patients with metastatic disease have a dismal outcome with 5-year survival rates of only 15–25% (Linabery & Ross, 2008). In fact, the presence of metastatic disease remains the single-most powerful predictor of outcome in ES (Bernstein et al, 2006). Patients with early relapse following therapy have a similarly poor outcome, most commonly due to the development of pulmonary or bone metastases. Numerous studies have begun to dissect mechanisms of transformation by EWS–ETS fusion oncoproteins, which function as chimeric transcription factors (Riggi & Stamenkovic, 2007). While revealing important insights into disease pathogenesis and establishing new molecular tools for diagnosis, these findings have not significantly impacted on treatment and outcome. First, EWS–ETS chimeras are challenging to drug, although potential downstream pathways may be more tractable for targeting (Braunreiter et al, 2006; Prieur et al, 2004; Smith et al, 2006). Second, EWS–ETS proteins are expressed in both localized and metastatic disease, and thus do not on their own account for metastatic behaviour (Ginsberg et al, 1999). Thus, there is an urgent need to identify specific biologic drivers orchestrating the process of metastasis in ES, and to evaluate novel therapeutics potentially targeting the involved pathways, either alone or in combination with standard-of-care chemotherapeutic agents.

There are surprisingly few reports relating to molecular mediators of metastasis in ES (Bennani-Baiti et al, 2010; Krishnan et al, 2006; Sainz-Jaspeado et al, 2010). We previously focused on the capacity of ES cells to suppress *anoikis*, or programmed cell death after detachment from the extracellular matrix (Frisch & Francis, 1994), to screen for candidate metastasis drivers in ES. Resistance to *anoikis* is critical for cancer cells to survive under anchorage independent conditions such as the circulation or the lymphatics, prior to establishment of overt metastases (Simpson et al, 2008). To model *anoikis* resistance *in vitro*, cell lines are cultured in suspension rather than conventional monolayers. *In vitro* survival under such conditions correlates strongly with *in vivo* metastasis of the same cell lines in immunodeficient mice (Douma et al, 2004). We previously found that TC32 and TC71 ES cell lines survive as multicellular spheroids in non-adherent suspension cultures (Kang et al, 2007; Lawlor et al, 2002). This correlated with increased activation of the PI3 kinase (PI3K)-Akt pathway but not Ras-ERK1/2, and reduced sensitivity to multiple cytotoxic agents compared with conventional monolayer cultures (Kang et al, 2007). In addition, the ERBB4 (HER4) receptor tyrosine kinase, a member of the epidermal growth factor receptor (EGFR) family, was preferentially activated under anchorage independent conditions. This was not associated with changes in ERBB4 protein expression, but correlated with both PI3K-Akt activation and chemoresistance in these cell lines (Kang et al, 2007). These findings suggest a potential link between ERBB4 and advanced disease in ES. However, a role for ERBB4 in metastatic progression has not been previously reported.

We now report a novel function for ERBB4 as a metastatic driver in ES. ERBB4 is overexpressed in ES cell lines derived from advanced tumours as well as in human metastatic ES lesions, and leads to activation of PI3K-Akt, focal adhesion kinase (FAK), and the Rac1 GTPase, a well-established mediator of cell migration and invasion. ERBB4 expression is directly correlated with increased *in vivo* metastasis of ES cell lines, and is upregulated in metastatic ES tumours. These studies provide new insights into the biology of ERBB4 in ES, and independently validate ERBB4 as a metastasis associated factor in this disease.

## RESULTS

### ERBB4 is overexpressed in cell lines derived from ES

**Figure 1:**
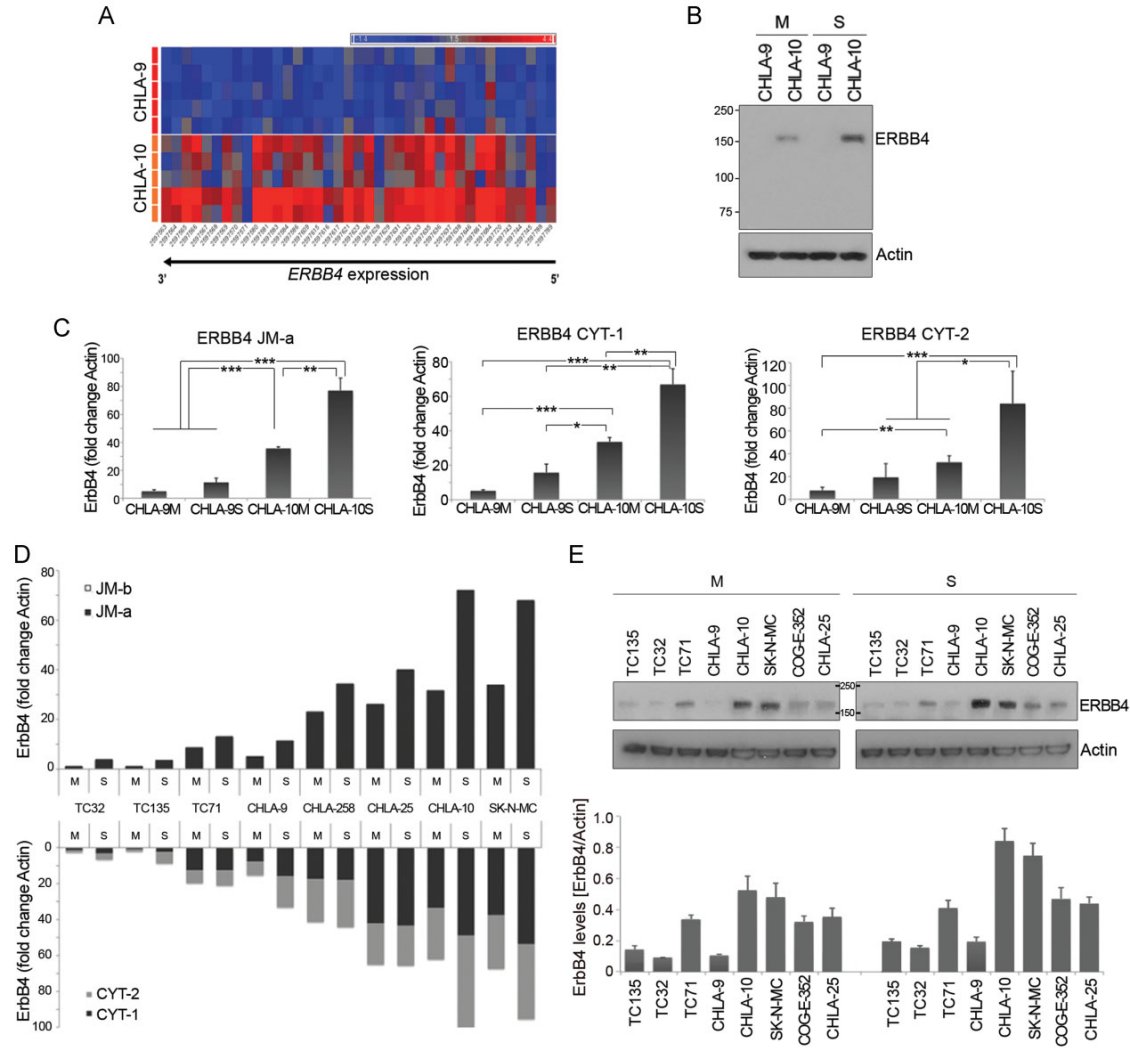
ERBB4 protein is overexpressed in cell lines derived from chemoresistant or metastatic ES Heat-map reveals increased *ERBB4* expression across most exons in CHLA-10 compared to CHLA-9 ES cells; data show *n* = 5 repetitions.ErbB4 expression levels were analyzed by Western blot in CHLA-9 and CHLA-10 cells cultured under M or S conditions. Actin was used as a loading control. One of three representative experiments is shown.ERBB4 isoforms expression was analyzed by qPCR in CHLA-9 and CHLA-10 cells grown as M or S, using ERBB4 TaqMan probes. Graphs represent mean ± SD of three independent experiments with two replicates each (*p < 0.05; **p < 0.01; ***p < 0.005). Specific numerical *p*-values for these and all subsequent experiments are itemized in Supporting Information Table S6.ERBB4 isoforms expression in samples from eight ES cell lines grown as M or S was evaluated by TaqMan qPCR. Top depicting JM-a and JM-b, and bottom CYT-1 and CYT-2 expression; *n* = 3 in duplicate.ERBB4 expression levels were analyzed by Western blotting in a panel of ES cell lines. Actin was used as a loading control. One of three representative experiments is shown.

To screen for potential markers of aggressive disease in ES, we compared gene expression profiles (GEPs) in CHLA-10 *versus* CHLA-9 ES cell lines. The latter was established from an untreated primary ES at the time of diagnosis, while the CHLA-10 cell line was established from a recurrence in the same patient after four cycles of induction chemotherapy with cisplatin, doxorubicin, cyclophosphamide and etoposide (Batra et al, 2004). GEPs generated using Affymetrix Human Exon (HuEx) arrays revealed a number of cell line specific differences. Forty-six genes were found to be significantly differentially expressed (with at least sevenfold altered expression), with 40 genes significantly upregulated and 6 genes significantly downregulated in CHLA-10 compared to CHLA-9 cells (Supporting Information Fig S1A). The full list of all genes, their encoded proteins, and known functions and relation to cancer can be found in Supporting Information Table S1. Among the upregulated genes in our dataset, transcripts encoding ERBB4 were ∼10-fold increased in CHLA-10 compared to CHLA-9 cells (Fig 1; *p* = 0.015). We focused on ERBB4, given the previous link between activation of this tyrosine kinase and *anoikis* suppression in ES (Kang et al, 2007). Western blotting demonstrated dramatically higher expression of the full-length 180 kDa ERBB4 species in CHLA-10 compared to CHLA-9 cells under conventional monolayer (M) culture conditions, which was further enhanced in non-adherent suspension (S) cultures of the same cell lines (Fig 1). We then tested whether the observed ERBB4 induction was transcriptionally regulated. *ERBB4* transcripts are known to undergo alternative splicing to generate different juxtamembrane (JM-a and JM-b) and cytoplasmic (CYT-1 and CYT-2) isoforms of full-length ERBB4 (Junttila et al, 2003). We therefore used previously described TaqMan quantitative RT-PCR (qRT-PCR) assays (Junttila et al, 2003) to specifically identify *ERBB4* splice forms that are expressed in CHLA-9 and CHLA-10 cells. This revealed increased expression of JM-a/CYT-1 and JM-a/CYT-2 transcripts in CHLA-10 compared to CHLA-9 cells, and both were increased in S compared to M cultures for each cell line (Fig 1); there was no evidence for expression of the JM-b splice form (data not shown). We then extended this analysis to a larger panel of ES cell lines. TaqMan qPCR revealed variable JM-a/CYT-1 and JM-a/CYT-2 ERBB4 transcript expression in M cultures, which was generally enhanced in S cultures of the same cell lines (Fig 1). TC71 only showed modest increase in mRNA levels when cultured in suspension. Again the JM-b splice form was not observed in any of these cell lines (Fig 1). Immunoblotting confirmed that ERBB4 protein levels correlated with increased JM-a/CYT-1 and JM-a/CYT-2 transcript expression (Fig 1). The highest overall expression was observed in CHLA-10, SK-N-MC, COG-E-352, CHLA-25 and TC71 cell lines. Each of these cell lines was derived from post-chemotherapy ES tumours (Supporting Information Table S2), further highlighting a link between ERBB4 induction and aggressive disease in ES.

Processing of the full-length JM-a/CYT-1 or JM-a/CYT-2 isoforms by tumour necrosis factor-α-converting enzyme (TACE) and γ-secretase is known to generate an 80 kDa (p80) ERBB4 soluble intracellular fragment that undergoes nuclear translocation (Lee et al, 2002; Rio et al, 2000); p80 expression is associated with poor survival in breast cancer models (Junttila et al, 2005). However, we failed to identify differences in the ERBB4 intracellular form in CHLA-9 *versus* CHLA-10 cell lines; moreover, no differences in nuclear ERBB4 expression were observed following subcellular fractionation, even after treatment with the phorbol ester, PMA, known to activate TACE (Zhang et al, 2001) (data not shown). Genome-wide copy number analysis using Affymetrix SNP6.0 arrays failed to reveal *ERBB4* copy-number aberrations in any of the cell lines analyzed (Supporting Information Fig S1B). Moreover, sequencing of *ERBB4* in CHLA-9 and CHLA-10 cells did not detect any *ERBB4* mutations (data not shown). Therefore the mechanism of *ERBB4* mRNA induction in ES cell lines remains unknown. Taken together, our data indicate full-length ERBB4 protein is overexpressed in cell lines derived from post-chemotherapy ES, and that this occurs through transcriptional upregulation of *ERBB4* JM-a/CYT-1 and JM-a/CYT-2 splice forms.

### ERBB4 activates the PI3K-Akt pathway under diverse forms of cell stress

**Figure 2:**
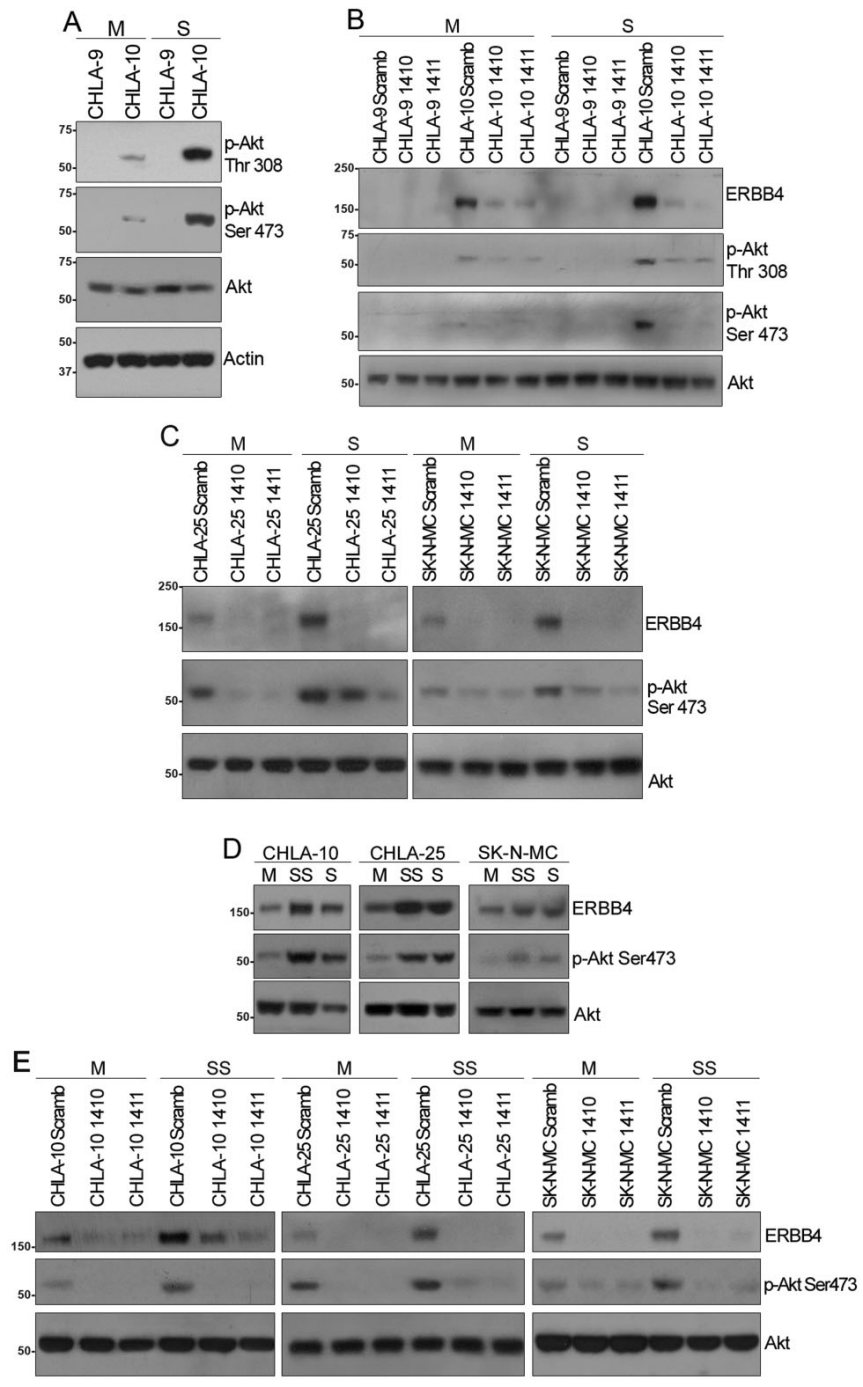
ERBB4 regulates PI3K-Akt in metastatic ES cell lines under diverse forms of cell stress **A.** Akt phosphorylation on Ser-473, Thr-308 or total Akt expression levels were evaluated by immunoblotting in CHLA-9 and CHLA-10 cells grown under M or S conditions. Blots are representative of *n* = 3 independent experiments.**B,C.** Targeting ERBB4 with 1410 and 1411 shRNA constructs reduced Akt phosphorylation in CHLA-10, CHLA-25 and SK-N-MC metastatic ES cells. One of three representative experiments is shown.**D.** Metastatic ES cell lines were growth as monolayer in serum-stimulated (M), serum-starved (SS) or spheroid (S) conditions for 24 h. ERBB4, p-Akt and total Akt levels were evaluated by Western blot; *n* = 3 independent experiments.**E.** ERBB4 kd reduced Akt activation in metastatic ES cell lines growth as M or SS conditions; *n* = 3 independent experiments.

CYT-1 contains a PI3K binding motif, which is required for PI3K-Akt activation and regulation of cell survival by ERBB4 (Elenius et al, 1999). We therefore assessed if elevated ERBB4 expression is associated with PI3K-Akt stimulation. Indeed, increased ERBB4 expression correlated with augmented Akt activation, as phospho-Thr-308 and Ser-473 Akt levels were both dramatically increased in CHLA-10 compared to CHLA-9 cells, particularly in S cultures (Fig 2), which correlates with our previous findings in ES cell lines (Kang et al, 2007). To validate the contribution of ERBB4 to the prosurvival PI3K-Akt pathway, we used short hairpin RNAs (shRNAs) for stable ERBB4 knockdown (kd). Two independent shRNA targeting constructs (designated 1410 and 1411) strongly downregulated ERBB4 protein expression in both M and S cultures of CHLA-10 cells (Fig 2). These shRNAs also dramatically reduced ERBB4 tyrosine phosphorylation (Tyr-P) levels in CHLA-10 cells, but did not affect Tyr-P of other EGFR family proteins, as assessed using R&D Human phospho-RTK Proteome arrays (Supporting Information Fig S2A and S2B). ERBB4 kd strongly reduced Akt activation in both M and S cultures of CHLA-10 cells, but particularly in the latter (Fig 2), as well as in S cultures of two other high ERBB4-expressing ES cell lines, CHLA-25 and SK-N-MC (Fig 2). Rescue of ERBB4 expression in 1410 or 1411 kd cells using an ERBB4 construct engineered to be resistant to knockdown through silent mutations in the ERBB4 shRNA targeting region restored ERBB4 expression and Akt activation in ERBB4 kd cells (Supporting Information Fig S2C), validating the specificity of these shRNAs. ERBB4 kd significantly increased apoptosis in the high ERBB4-expressing ES lines, CHLA-10, CHLA-25 and SK-N-MC, as demonstrated by increased cleaved PARP (c-PARP) levels (Supporting Information Fig S2D). Induction of apoptosis was additionally confirmed by propidium iodide staining and FACS analysis in CHLA-10 cells grown as M and S cultures (Supporting Information Fig S2E; *p* < 0.005). We then assayed ES cells for expression of known ERBB4 ligands (Revillion et al, 2008), revealing variable expression of heparin binding EGF-like growth factor (HB-EGF), neuregulin 1 and 2 (NRG-1 and NRG-2), and amphiregulin (ARG) (Supporting Information Fig S2F), while betacellulin (BTC) was not expressed (data not shown). Treatment of CHLA-10 S cultures with HB-EGF, NRG-1 or NRG-2 did not further increase ERBB4 Tyr-P (Supporting Information Fig S2G), or Akt Ser-473 phosphorylation, (Supporting Information Fig S2H) since ERBB4 and Akt were already strongly activated under these conditions. Together, these findings show that high ERBB4 protein expression is associated with PI3K-Akt activation and increased survival in ES cell lines.

Since detachment from the extracellular matrix, such as during enforced growth in suspension, is a form of cell stress (Ng et al, 2012), we wondered if ERBB4 induction might be part of a more generalized stress response. We therefore tested if ERBB4 expression is also induced under a second form of prototypical cell stress, namely serum starvation (SS). Indeed, compared to M cultures, ERBB4 protein expression was further upregulated under SS conditions in CHLA-10, CHLA-25 and SK-N-MC high ERBB4-expressing ES cell lines, which strongly correlated with increased Akt activation (Fig 2). ERBB4 kd almost completely blocked Akt activation under SS conditions in each cell line (Fig 2). Low ERBB4-expressing CHLA-9 ES cells were then transduced with lentiviral constructs encoding full-length ERBB4 or with control vector (pLEOC) alone, and cells were grown under M, S or SS conditions as above. ERBB4 overexpression in these cells strongly activated Akt in each culture condition (Supporting Information Fig S2I), confirming a direct correlation between ectopic ERBB4 expression and Akt activation in ES cells. Taken together, these findings indicate that ERBB4 overexpression in ES cells regulates the PI3K-Akt pathway under diverse forms of cell stress.

### ERBB4 increases migration and invasion of ES cell lines *in vitro*

**Figure 3:**
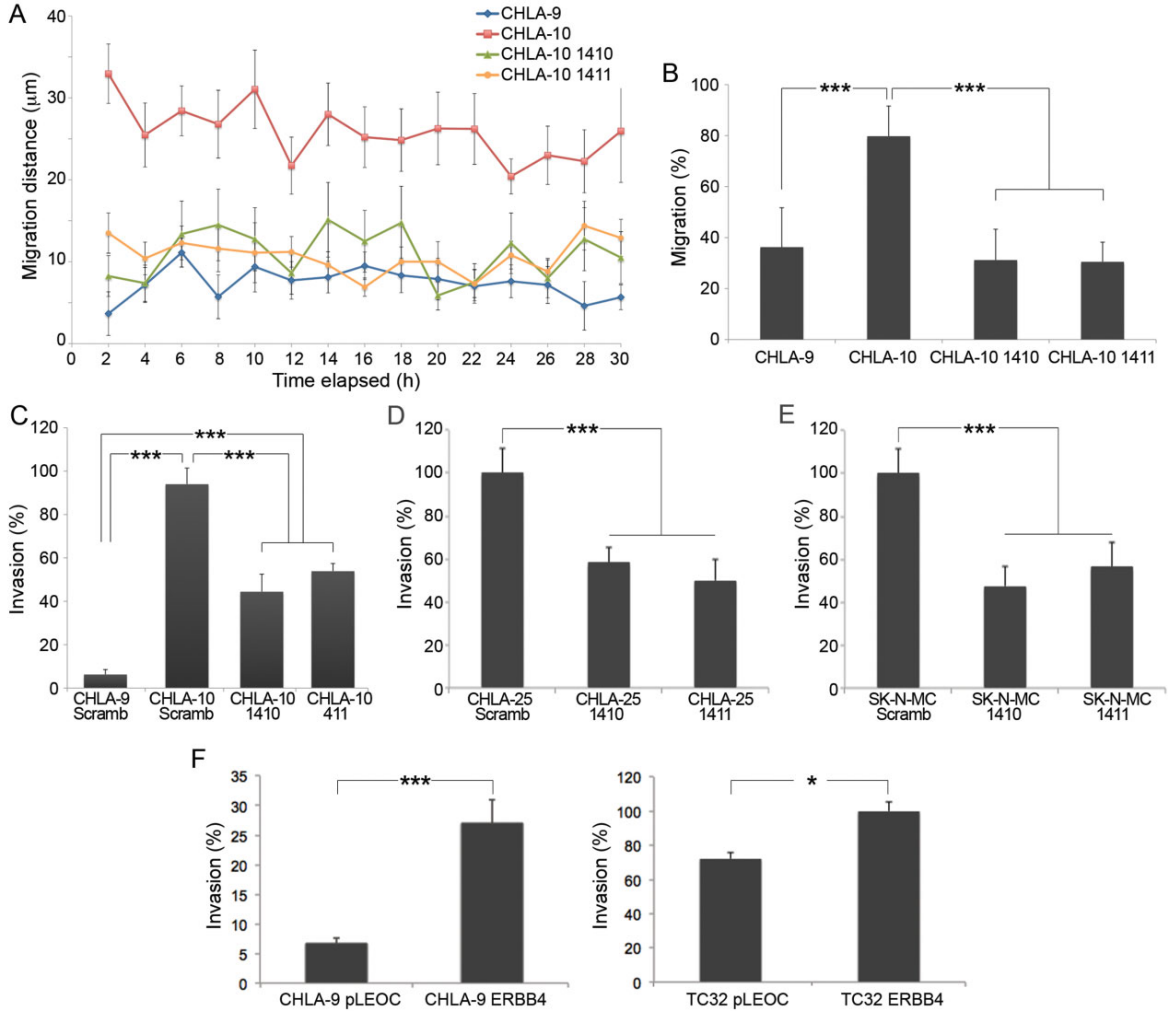
Knockdown of ERBB4 impairs migration and invasion in cell lines derived from chemoresistant or metastatic ES **A.** Confluent monolayers of CHLA-9 and CHLA-10 ES cells transduced with 1410 and 1411 ERBB4 shRNA constructs were wound-scratched and allowed to migrate for 30 h. The graph shows migration distance *vs*. time values ± SD of six independent experiments.**B–E.** Uncoated (B), or Matrigel-coated transwells (C–E) were used to analyze the contribution of ERBB4 to B, the cell migration, and C–E, invasion processes in control, or in ERBB4 kd CHLA-9, CHLA-10, SK-N-MC and CHLA-25 cells. Graphs represent mean ± SD of four independent experiments; ***p < 0.005.**F.** Overexpression of ERBB4 in CHLA-9 and TC-32 ES cells significantly increases cell invasion; *n* = 3, *p < 0.05, ***p < 0.005.

Since increased ERBB4 expression was observed in ES cell lines derived from post-chemotherapy ES, we tested whether this kinase might also regulate migration and invasion of ES cells, two essential components of the metastatic process. Using scratch assays we found that high ERBB4-expressing CHLA-10 cells migrated significantly faster than low-expressing CHLA-9 cells, but this was markedly reduced in CHLA-10 1410 and 1411 ERBB4 kd cells (Fig 3 and Supporting Information Fig S3A; *p* < 0.005). Lapatinib (http://www.cancer.gov/cancertopics/druginfo/fda-lapatinib), a pan-ERBB inhibitor that decreased ERBB4 Tyr-P in CHLA-10 cells (Supporting Information Fig S3B), also significantly reduced ES cell migration, but to a lesser degree than ERBB4 kd (Supporting Information Fig S3A). Invasive capacity of metastatic ES cell lines with or without ERBB4 kd was next examined using Matrigel-coated Boyden chambers. Cell invasion was enhanced in CHLA-10 compared to CHLA-9 cells, and again was significantly reduced by ERBB4 kd in each high ERBB4-expressing cell line tested, CHLA-10, SK-N-MC and CHLA-25 (Fig 3). Differences in cell migration/invasion were not due to reduced cell survival following ERBB4 kd, since the rate of cell death over the time-course of the experiments (16–24 h) was minor and accounted for only 5–15% of cell death in all three lines analyzed (Supporting Information Fig S3C). Cell invasion was rescued in ERBB4 kd cells transduced with non-targeting ERBB4 constructs (Supporting Information Fig S3D), further supporting the specificity of the ERBB4-mediated invasive phenotype. ERBB4 overexpression in low ERBB4-expressing CHLA-9 and TC32 ES cell lines also variably but significantly increased invasion (Fig 3; *p* < 0.05–0.005) and migration (Supporting Information Fig S3E; *p* < 0.05–0.005). These data indicate that ERBB4 overexpression alone increases *in vitro* markers of aggressive behaviour in ES cells.

### ERBB4 regulates Rac1 GTPase activity and cell spreading in ES cell lines

**Figure 4:**
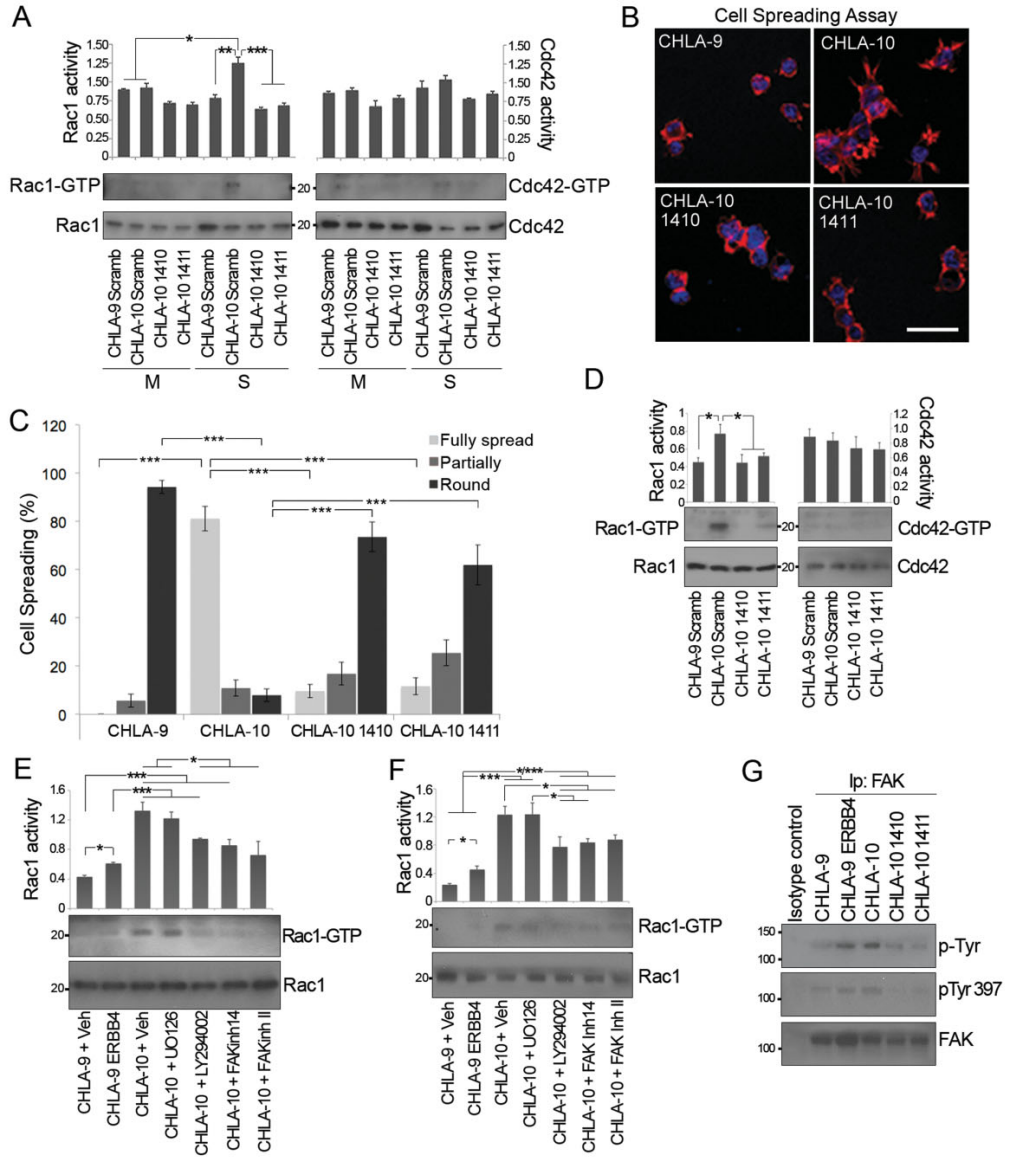
ERBB4 regulates Rac1 GTPase activity through PI3K and FAK downstream pathways **A.** Rac1 and Cdc42 GTPase activities were measured by pull-down assays in CHLA-9 and CHLA-10 Scramb, or ERBB4 kd cells growth as M or S. Data shown are representative of *n* = 3 experiments; *p < 0.05; **p < 0.01; ***p < 0.005.**B.** Phalloidin (red) and Hoechst nuclear staining (blue) of CHLA-9, CHLA-10 and ERBB4 kd CHLA-10 cells are shown.**C.** Graph shows quantification of cell spreading; at least 25 cells were scored per condition. Values represent percentage of the mean ± SD; *n* = 3, ***p < 0.005.**D.** Active Rac1 and Cdc42 (GTP-bound) levels were evaluated in control CHLA-9 and CHLA-10 cells, or in CHLA-10 ERBB4 kd cells 2 h after spreading assays. Rac1 and Cdc42 from total lysates were used as loading controls. Data shown are representative of three independent experiments; *p < 0.05.**E,F.** GTP-bound Rac1 levels were evaluated by pull-down assays in CHLA-9 control or CHLA-9/ERBB4 cells, and in CHLA-10 cells treated with different inhibitors. Cells were growth under: (E) anchorage independent (S), or (F) cell spreading conditions. Rac1 from total lysates was used as a loading control. Graphs represent mean ± SD of three independent experiments; *p < 0.05, ***p < 0.005.**G.** Total p-Tyr and FAK Tyr397 phosphorylation were assessed by immunoprecipitation in CHLA-9, CHLA-9/ERBB4, CHLA-10 and CHLA-10 ERBB4 kd cells, during cell spreading assays. Lysates were immunoprecipitated for endogenous FAK, and immunoblotted with p-Tyr, FAK Tyr397 or FAK Abs. One of three representative experiments is shown.

To evaluate how ERBB4 enhances cell motility, we analyzed members of the Rho GTPase family, which control dynamic reorganization of the actin cytoskeleton to facilitate migratory and invasive capacity (Stonecypher et al, 2006), in CHLA-10 *versus* CHLA-9 cell lines. Using affinity-based pull-down assays, there was no evidence for RhoA activation in either cell line (data not shown). Similarly, Cdc42 was not significantly activated in either M or S cultures of CHLA-9 and CHLA-10 cells (Fig 4, right panels). However, while Rac1 GTPase activity was minimal in CHLA-9 and CHLA-10 M cultures (Fig 4, left panels), Rac1 activity was significantly increased in S compared to M cultures of CHLA-10 cells (Fig 4; *p* < 0.05). Moreover, activation was abrogated in ERBB4 kd CHLA-10 cells in suspension (Fig 4). We next examined the contribution of ERBB4 to cell spreading, known to be regulated by Rac1 and Cdc42 Rho GTPases (Price et al, 1998). Using TRITC-phalloidin staining as previously described (Price et al, 1998), we observed that by 2 h after seeding, ∼90% of CHLA-9 cells remained round, in striking contrast to CHLA-10 cells, of which ∼80% showed morphologic features of cell spreading (Fig 4; *p* < 0.005). Cell spreading led to marked Rac1 activation in CHLA-10 cells but not in CHLA-9 cells, while Cdc42 was not active in either cell line (Fig 4). ERBB4 kd significantly reduced spreading by ∼70% in CHLA-10 cells (Fig 4; *p* < 0.005), and almost completely blocked Rac1 activation (Fig 4; see 1410 and 1411 lanes; *p* < 0.05), reducing levels to those of CHLA-9 cells. We next assessed Rac1 GTPase activity in CHLA-9 cells overexpressing ERBB4 (CHLA-9/ERBB4). CHLA-9/ERBB4 cells showed a modest but reproducibly significant increase in Rac1 activation compared to vector control CHLA-9 cells, both in S cultures (Fig 4; *p* < 0.05) and during cell spreading (Fig 4; *p* < 0.05). These data provide compelling evidence that ERBB4 specifically activates Rac1 during anchorage independent growth and cell spreading, in keeping with the ability of ERBB4 to enhance invasiveness of ES cells.

### ERBB4 regulates Rac1 GTPase activity through PI3K-Akt and FAK pathways

In addition to specific guanine nucleotide exchange factors (GEFs), Rac1 activity is also regulated by PI3K (Marcoux & Vuori, 2003) and FAK (Chang et al, 2007). CHLA-10 cells were incubated with the LY-294002 PI3K inhibitor, two FAK inhibitors, FAKinh14 or FAKinhII, and the U0126 MEK inhibitor, as described in the Materials and Methods Section. Inhibition of Akt, ERK1/2 and FAK phosphorylation was confirmed using phospho-specific antibodies (Supporting Information Fig S4A). While U0126 had no effect on Rac1 activation, both LY294002 and FAKinh14 or FAKinhII strongly blocked Rac1 GTPase activity in CHLA-10 cells under S conditions (Fig 4) or during cell spreading (Fig 4). Reduced Rac1 activation following treatment with the different inhibitors was not due to reduced cell survival (Supporting Information Fig S4B). These data strongly support roles for both PI3K and FAK in regulating Rac1 activation in metastatic ES cells.

To further explore potential links between ERBB4 and FAK activation in ES cells, we immunoprecipitated FAK and immunoblotted for total Tyr-P and FAK Tyr-397 phosphorylation (as markers of FAK activation) in CHLA-9, CHLA-9/ERBB4, CHLA-10 and CHLA-10 ERBB4 kd cells. CHLA-10 displayed significantly higher FAK activity compared to CHLA-9 cells (Fig 4; Supporting Information Fig S4C, *p* < 0.005), and ERBB4 overexpression in CHLA-9 cells moderately increased FAK activation compared to CHLA-9 control cells (Fig 4; Supporting Information Fig S4C). ERBB4 kd significantly reduced both total Tyr-P and Tyr-397 phosphorylation of FAK in CHLA-10 cells (Fig 4; Supporting Information Fig S4C, *p* < 0.05), supporting a role for ERBB4 in regulating FAK activity in ES. Notably, LY294002 had no apparent effects on FAK activation, and FAK inhibitors only partially reduced Akt phosphorylation (Supporting Information Fig S4A); therefore the interplay between these pathways remains unclear.

**Figure 5:**
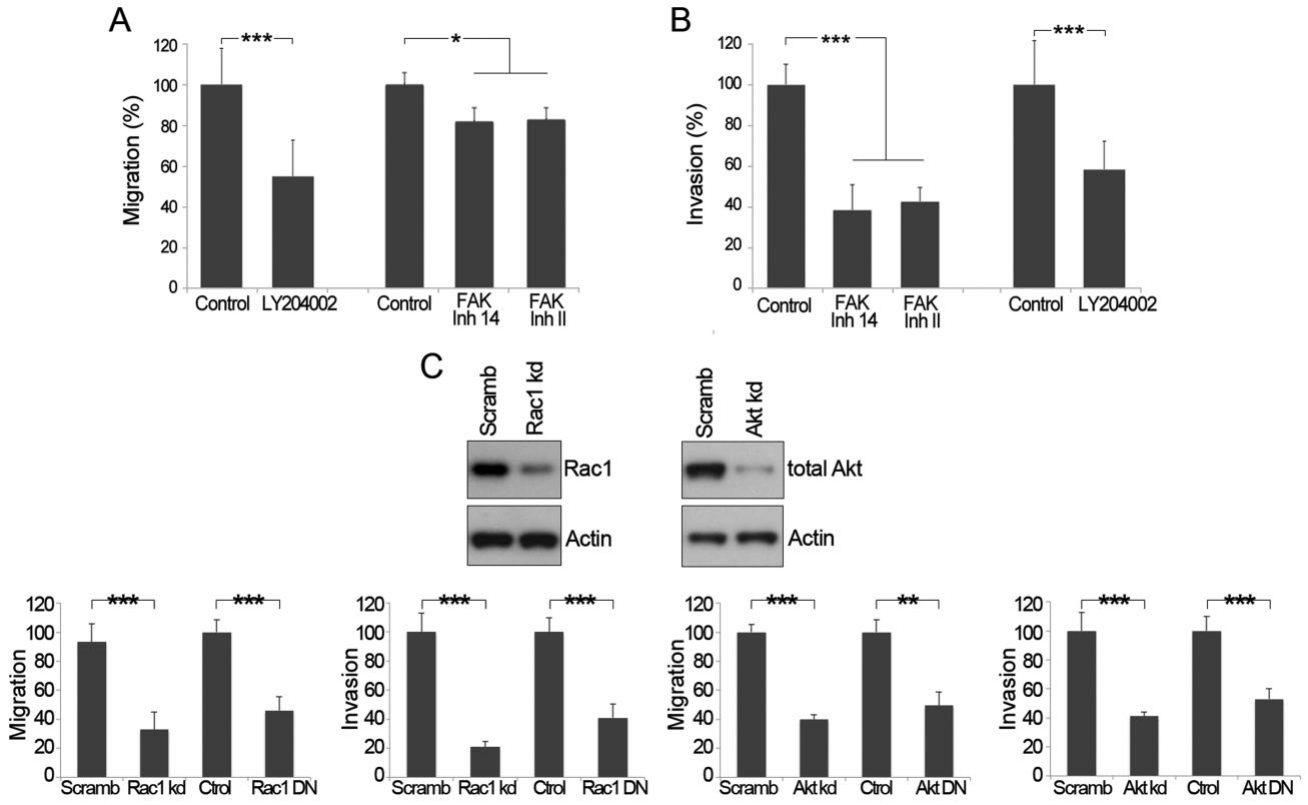
ERBB4 regulates cell migration and invasion through PI3K and FAK pathways **A,B.** The contribution of PI3K and FAK to the cell migration, and invasion processes was analyzed in control CHLA-10 cells, or in CHLA-10 cells treated with LY294002, and FAKinhII or FAKinh14 inhibitors. Graphs represent mean ± SEM of three independent experiments; *p < 0.05, ***p < 0.005.**C.** Knockdown of Rac1 and Akt, or transfection with Rac1 or Akt DN constructs reduced migration and invasion in CHLA-10 cells. Graphs represent mean ± SD of *n* = 3 experiments; **p < 0.01, ***p < 0.005.

Finally, we assessed effects of PI3K and FAK inhibition on ES cell migration and invasion. Inhibition of either PI3K or FAK moderately but significantly reduced migration and strongly inhibited invasion in CHLA-10 cells *in vitro* (Fig 5). We then tested whether downstream effectors of PI3K and FAK, namely Akt and Rac1, were involved in ERBB4 effects on cell motility. We therefore performed siRNA knockdown of Akt 1 and 2 or Rac1 in CHLA-10 cells, as well as expression of dominant negative Akt (Zhang et al, 2003) or Rac1 constructs (Nobes & Hall, 1999). As clearly shown in Fig 5, both approaches provide compelling evidence that reducing Akt or Rac1 levels or activity markedly reduces both migration and invasion of high ERBB4 expressing CHLA-10 cells. Together, these data indicate that ERBB4 regulates Rac1 GTPase activity and ES cell migration and invasion through both PI3K and FAK pathways.

### ERBB4 increases ES metastasis formation *in vivo*

**Figure 6:**
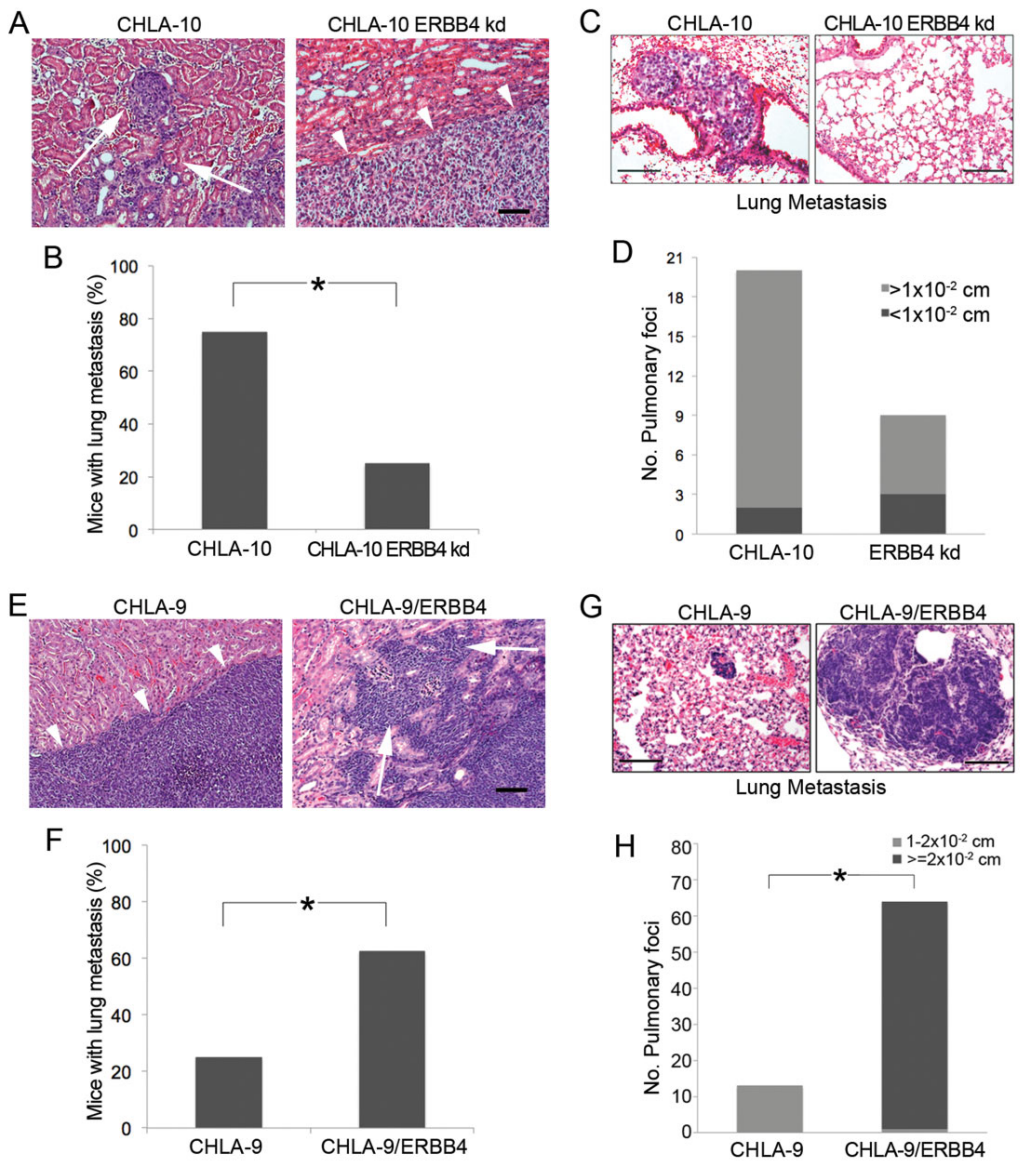
ERBB4 contributes to invasion and metastasis formation *in vivo* **A,E.** Side-by-side view of low power H&E stained primary tumours from CHLA-10 and CHLA-10 ERBB4 kd (A), and CHLA-9 and CHLA-9/ERBB4 (E) implanted mice. Arrows in A and E point to highly infiltrative borders with direct invasion into adjacent normal kidney, whereas arrowheads point to the so-called “pushing” non-invasive borders.**B,F.** The percentage of animals from each of these groups harbouring metastatic lesions was quantified (*n* = 8 animals/condition; *p < 0.05).**C,G.** Representative H&E stained images of: (C) CHLA-10 and CHLA-10 ERBB4 kd, and (G) CHLA-9 and CHLA-9/ERBB4 pulmonary metastasis are shown.**D,H.** The number and size of lung metastatic lesions from each of these groups were quantified; *p < 0.05.

**Figure 7:**
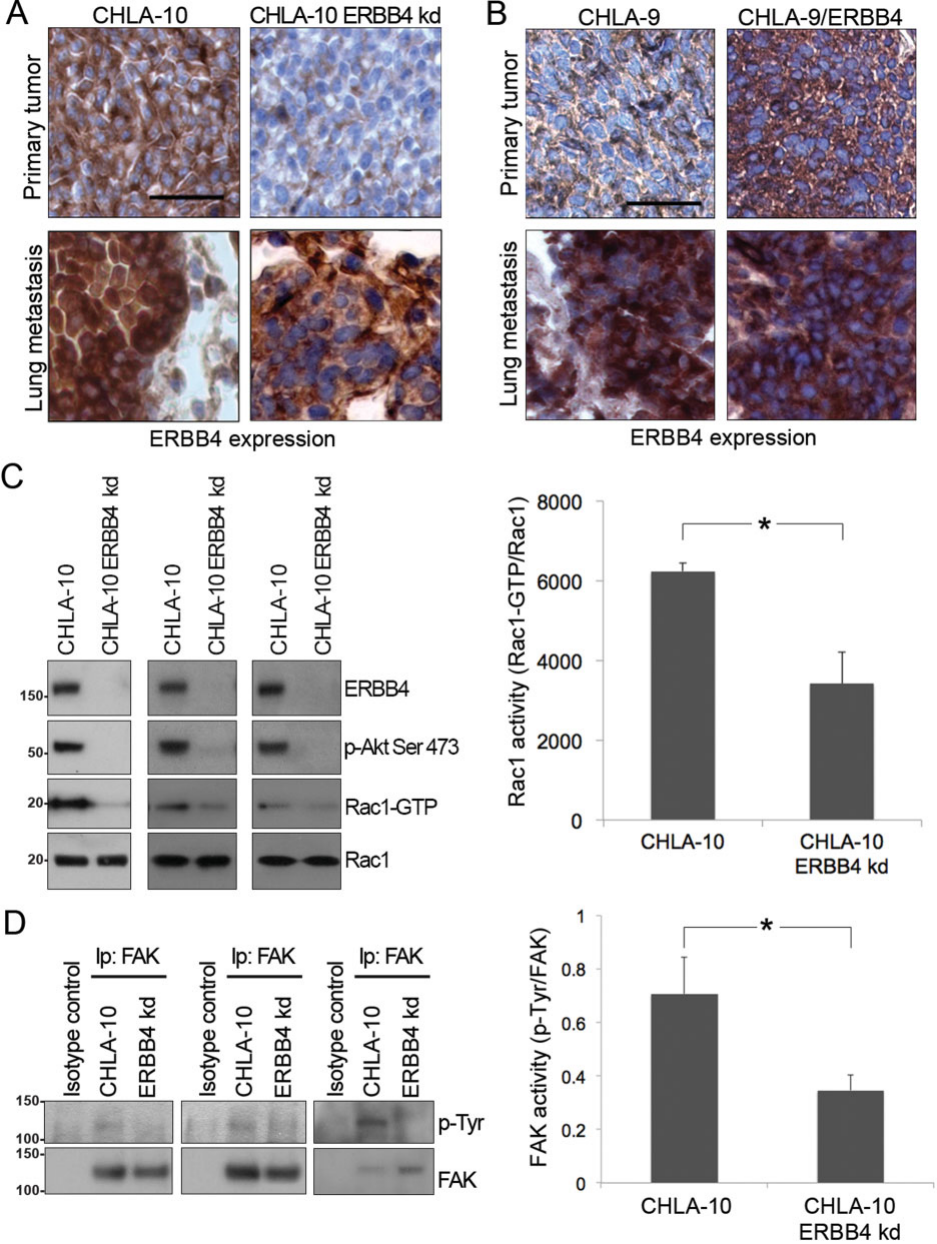
ERBB4 regulates Akt, Rac1 and FAK activation *in vivo* **A,B.** Representative images of IHC staining for ERBB4 in primary tumours (upper panels), or metastatic lesions (lower panels) of CHLA-10, CHLA-10 ERBB4kd, CHLA-9 or CHLA-9/ERBB4 tumours are shown.**C.** GTP-bound Rac1 levels were evaluated by pull-down assays in tumours from CHLA-10 and CHLA-10 ERBB4 kd mice; *n* = 3 mice per condition. Total Rac1, ERBB4 and p-Akt Ser-473 were additionally evaluated by immunoblotting. Graph represents Rac1 activity ± SEM; *n* = 3, *p < 0.05.**D.** Lysates were immunoprecipitated for endogenous FAK, and immunoblotted with p-Tyr or FAK Abs. An isotype-matched antibody was used as a negative control. Graph represents FAK activity ± SEM; *n* = 3, *p < 0.05.

The above studies predict that ERBB4 may regulate ES metastasis *in vivo*. To test this we used the previously described mouse renal subcapsular implantation (SCI) model (Cheng et al, 2010). This model exhibits macroscopic, high-volume metastases of diverse human tumour cell lines more rapidly than most subcutaneous or orthotopic models, likely due to high vascularization of the renal subcapsule graft site (Nakamura et al, 2011; Watahiki et al, 2011). We adapted the SCI model to monitor growth of human ES cell lines *in vivo*, as described in the Materials and Methods Section. As shown in Supporting Information Fig S5A, both scrambled control CHLA-10 and ERBB4 kd cells formed large tumours under the renal capsules of implanted mice with the morphologic features of ES, and no differences in tumour sizes were observed (data not shown). However, CHLA-10 control cells demonstrated highly infiltrative borders with direct invasion into adjacent normal kidney (Fig 6, arrows), while CHLA-10 ERBB4 kd cells exclusively grew with so-called “pushing” non-invasive borders into adjacent normal kidney (Fig 6, arrowheads). ERBB4 knockdown tumours also showed extensive areas of necrosis (Supporting Information Fig S5A; see arrows). Moreover, while 75% of mice implanted with scrambled control CHLA-10 cells developed lung metastases, the rate of metastatic spread to lungs in mice implanted with CHLA-10 ERBB4 kd cells was significantly reduced to <25% (Fig 6; *p* < 0.05). In addition, ERBB4 kd induced marked decreases in both the average number and size of pulmonary foci (Fig 6). High ERBB4 expression in control CHLA-10 primary implantation site tumours was confirmed by immunohistochemistry (IHC), and a dramatic reduction of ERBB4 protein expression was seen in CHLA-10 ERBB4 kd implantation site tumours (Fig 7, upper panels). Interestingly, both CHLA-10 control and ERBB4 kd lung metastases displayed strong ERBB4 immunoreactivity (Fig 7, lower panels), suggesting that in the ERBB4 kd group, metastatic tumours formed from cells that escaped ERBB4 knockdown or re-expressed ERBB4 following shRNA silencing.

We then performed the converse experiment by implanting vector control or ERBB4 overexpressing CHLA-9 cells using the SCI model. Both cell lines formed large tumours at primary implantation sites, although CHLA-9 vector control tumours showed marked necrosis (Supporting Information Fig S5B). However, whereas CHLA-9 control cells showed pushing borders into adjacent normal kidney similar to CHLA-10 ERBB4 kd cells (Fig 6, arrowheads), remarkably, implanted CHLA-9/ERBB4 cells grew with a highly infiltrative pattern similar to CHLA-10 control cells (Fig 6, arrows), clearly demonstrating that ERBB4 induces an invasive phenotype *in vivo*. Notably, ERBB4 overexpression significantly increased the proportion of mice with lung metastases from ∼22 to >60% (Fig 6; *p* < 0.05), and strikingly increased the average number and size of metastatic tumours (Fig 6). Reduced ERBB4 expression was confirmed by IHC in CHLA-9 control implantation site tumours compared to strong ERBB4 immunostaining in both CHLA-9/ERBB4 implantation site and metastatic tumours (Fig 7).

To evaluate ERBB4 downstream signalling *in vivo*, we compared Akt, FAK and Rac1 activity in implantation site tumours isolated from mice implanted with CHLA-10 control *versus* CHLA-10 ERBB4 kd cells. In tumours isolated from each of three separate mice, ERBB4 kd led to almost complete abrogation of Akt phosphorylation and significantly reduced Rac1 (Fig 7; *p* < 0.05), and FAK activation (Fig 7; *p* < 0.05). This clearly demonstrates that ERBB4 regulates Akt, FAK and Rac1 activation *in vivo*. Taken together, these data strongly support an *in vivo* role for ERBB4 in facilitating increased metastatic capacity in ES.

### ERBB4 expression is increased in metastatic ES tumours and correlates with disease progression

**Figure 8:**
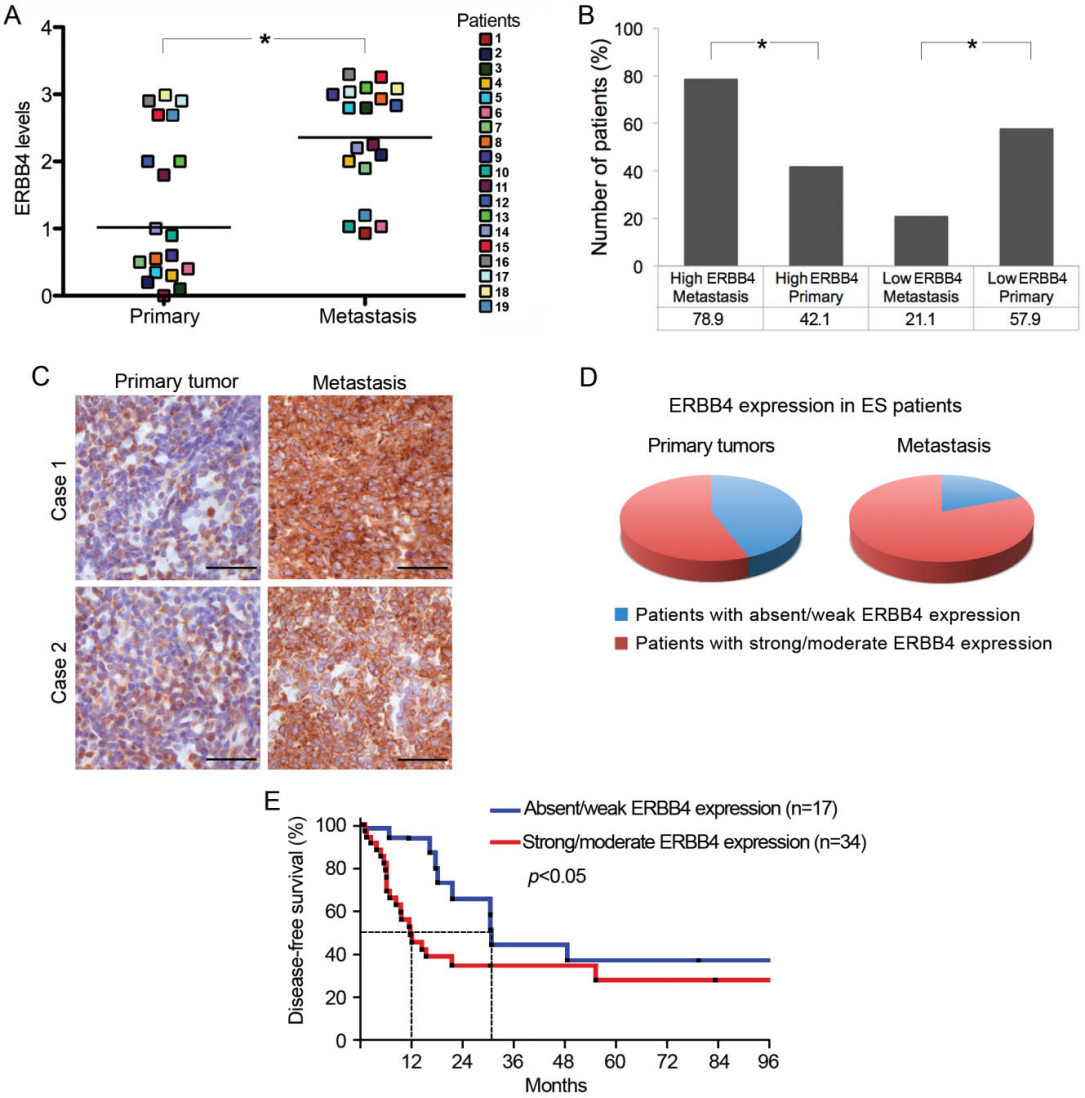
ERBB4 expression is elevated in metastatic tumour samples and correlates with disease progression in ES IHC was used to examined ERBB4 expression in 19 matched biopsies (primary tumours and metastatic sites) from the same patients; *p < 0.05.The graph shows the percentage of ES patients displaying weak/absent *versus* strong/moderate ERBB4 expression in localized *versus* metastatic lesions; *n* = 19; *p* < 0.05.Representative IHC images depicting ERBB4 staining in matched primary and the corresponding distant metastasis from two ES patients are shown. Scale bars = 50 µm.ERBB4 immunoreactivity was analyzed by IHC in a TMA containing *n* = 94 clinical samples. High ERBB4 expression in the metastatic lesions was found in 81.3% patients *versus* 57.7% patients in the primary sites; *p* < 0.05.Kaplan–Meier illustrating disease-free survival of ES patients, classified according to ERBB4 protein expression. Data show a significantly worse clinical outcome (*p* < 0.05) associated with high ERBB4 expression levels.

Given that ERBB4 suppresses *anoikis* and increases migration and invasion of ES cells *in vitro*, and enhances their metastatic capacity *in vivo*, we wondered whether ERBB4 expression correlates with metastatic disease in ES patients. We analyzed a cohort of 19 paired biopsies of primary and metastatic ES tumours from the same patients, as well as a tissue microarray (TMA) containing a further 94 (78 primary and 16 metastatic) non-paired ES tumours. IHC was used to assess ERBB4 protein expression as described (Kang et al, 2007). Patient characteristics are summarized in Supporting Information Table S3. ERBB4 quantification showed significantly higher ERBB4 expression in metastatic lesions *versus* patient-matched primary tumours (Fig 8; *p* < 0.05), with 78.9% of patients having high (strong/moderate) ERBB4 expression in metastatic lesions compared to 42.1% in the primary tumours (Fig 8; *p* < 0.05). The TMA revealed a significantly greater percentage of cases with high ERBB4 expression in the metastases compared to primary tumours (81.3% *vs*. 57.7%, respectively; Fig 8; *p* < 0.05). In 48 patients with available demographic data, high ERBB4 was not associated with clinic-pathological variables such as age, gender or primary tumour site, nor with ERBB4 nuclear, cytosolic or membranous localization (Supporting Information Table S4). Kaplan–Meier survival analysis demonstrated a direct correlation between high ERBB4 expression and poor outcome in ES patients (Fig 8; *p* < 0.05). The median disease-free survival time for patients with high ERBB4 levels was 12.2 months, whereas the corresponding time for patients with low ERBB4 immunoreactivity was 30.9 months (Fig 8, *p* < 0.05). These findings confirm a link between high ERBB4 expression and metastatic disease in ES patient samples.

## DISCUSSION

Outcomes for ES patients with metastatic disease have not changed significantly in the past two decades, and the majority of these patients die of their disease even with current multi-modality therapy. With the goal of identifying candidate proteins or pathways involved in ES progression, we found that ERBB4 is transcriptionally induced in ES tumour cell lines derived from chemoresistant or metastatic ES tumours, and that ERBB4 protein expression is elevated when ES cell lines are subjected to diverse stresses such as detachment from the extracellular matrix or serum deprivation. Furthermore, ERBB4 is overexpressed in metastatic compared to primary lesions in TMAs of ES tumours. ERBB4 overexpression activates a novel ERBB4-PI3K-Akt-FAK-Rac1 pathway that stimulates migration and invasion of ES cells, and confers metastatic capacity *in vivo*. A recent study reported ERBB4 upregulation in neuroblastoma cells *in vitro* under anchorage independent or serum starvation conditions, which was also associated with marked chemoresistance (Hua et al, 2012) Therefore, while epithelial malignancies utilize EGFR and HER2 for oncogenic signalling through ErbB family RTKs, tumours of neural or mesenchymal origin may instead preferentially activate ERBB4, although additional studies are necessary to rigorously test this possibility.

The role of ERBB4 in cancer remains controversial, with evidence for both oncogenic (Junttila et al, 2005; Prickett et al, 2009) and tumour suppressor activities (Das et al, 2010; Sartor et al, 2001). One possible explanation for this apparent dichotomy is that ERBB4 exists in four alternatively spliced isoforms, suggesting divergent activity of the different isoforms (Veikkolainen et al, 2011). Differences in the extracellular juxtamembrane (JM) domain encoding sequences arise from inclusion of exon 15 (JM-b) *versus* exon 16 (JM-a) in the mature transcript. The resulting JM-a isoform, but not JM-b, can be cleaved by the TACE protease and subsequently by a second protease, γ-secretase (Ni et al, 2001). This generates an 80 kDa active protein kinase that translocates to the nucleus to regulate differentiation through STAT5 signalling (Lee et al, 2002; Rio et al, 2000), and nuclear localization of this soluble intracellular domain is associated with poor survival in breast cancer models (Junttila et al, 2005). However, we did not detect evidence for nuclear ERBB4 protein accumulation in high ERBB4-expressing ES cells, even after treatment with PMA, which activates TACE (Zhang et al, 2001). Moreover, nuclear ERBB4 expression in clinical samples did not correlate with Ewing patient survival. Another ERBB4 isoform pair derives from differences in the cytoplasmic domain, based on the presence or absence of exon 26 in the coding sequence, designated as CYT-1 and CYT-2, respectively (Elenius et al, 1999). CYT-1, but not CYT-2, contains a PI3K binding motif, which is required for PI3K-Akt activation by ERBB4. CYT-1 is known to regulate cell survival and protein synthesis (Elenius et al, 1999), and its expression mediates resistance to nutrient deprivation in medulloblastoma (Ferretti et al, 2006). JM-a/CYT-1 and JM-a/CYT-2 were the only ERBB4 isoforms detected in ES cells; each were enhanced in metastatic/chemoresistant lines and further upregulated in anchorage independent cultures. Hence, CYT-1 upregulation may contribute to PI3K-Akt hyperactivation and cell survival observed under non-adherent conditions and serum starvation in ES cells. Binding of ERBB4 to its ligands has been linked to oncogenic activity in tumours including breast cancer (Mill et al, 2011). Our data using ligand stimulation suggest that ERBB4 may already be maximally activated under S conditions, such that additional ectopic ligand treatment is unlikely to further increase ERBB4 tyrosine phosphorylation and activation. Further experiments are necessary to determine if ERBB4 activation is ligand dependent or independent in ES cells. Genome-wide copy number analysis did not reveal *ERBB4* copy-number aberrations in any of the cell lines with ERBB4 overexpression, nor were *ERBB4* mutations detected. Therefore the mechanism of *ERBB4* transcriptional induction remains unknown. ERBB4 promoter methylation is one possibility, and we are exploring this experimentally. It is also interesting to note that the three ES cell lines with the lowest basal ERBB4 expression levels were also those with functional p53 status (Supporting Information Table S2), and so it will be important to determine if there is a link between p53 inactivation and stress-induced ERBB4 induction.

Our findings point to potential predictive value of ERBB4 overexpression as a biomarker for metastatic disease in ES. Although previous studies have shown associations between ERBB4 expression and different human cancers (Ferretti et al, 2006; Frey et al, 2010; Junttila et al, 2005; Kang et al, 2007; Maatta et al, 2006; Mill et al, 2011; Sartor et al, 2001), to date no links with tumour metastasis have been reported. Forced ERBB4 overexpression in non-metastatic CHLA-9 ES cells led to a highly infiltrative growth pattern into surrounding kidney tissue after renal SCI, similar to what was observed for implanted CHLA-10 cells, which high basal ERBB4 expression. Infiltrative borders were strongly associated with increased pulmonary metastasis. Conversely, ERBB4 kd completely blocked this phenomenon in CHLA-10 cells, and reverted the phenotype to the non-invasive well-demarcated borders of implanted control CHLA-9 cells, characteristic of cells of limited metastatic potential (Zlobec et al, 2009). These studies demonstrate that ERBB4 expression by itself is sufficient to promote invasive and metastatic potential in ES.

The signalling pathways underlying progression and acquisition of the metastatic phenotype are largely unknown. ERBB4 activation markedly activated Rac1, which has well-established links to cytoskeletal dynamics and cell migration, and which is implicated in cancer cell invasion and metastasis (Parri & Chiarugi, 2010). ERBB4-mediated Rac1 activation in ES cells was regulated through both PI3K and FAK, as inhibition of each significantly impaired Rac1 activity as well as migration and invasion. PI3K, which interacts directly with and is regulated by ERBB4 (Elenius et al, 1999), is required for Rac1 activation (Vega & Ridley, 2008), and PI3K activation supports metastasis in many tumour types (Ni et al, 2001). FAK is required for EGF-induced cell migration, and is a key integrator of RTK and integrin signalling pathways to regulate cell motility (Sieg et al, 2000). Moreover, FAK has been linked to the invasive phenotype of tumours in a kinase and Rac1-dependent manner (Hsia et al, 2003). We found that ERBB4 kd blocks Tyr-397 phosphorylation, the major autophosphorylation site of FAK (Schaller et al, 1994), as well as overall FAK activation under cell spreading conditions, an early step in tumour cell migration and invasion (Timar et al, 1996). However, inhibition of PI3K had no apparent effects on FAK activation, and blocking FAK only partially reduced Akt phosphorylation. This suggests that ERBB4 activates FAK upstream of PI3K-Akt, and that ERBB4 can activate PI3K through both FAK-dependent and independent mechanisms. Since ERBB4 kd blocks both FAK and PI3K-Akt, and inhibition of each pathway impairs migration and invasion, our findings point to a dual role for these proteins in ERBB4-mediated migration and invasion of ES cells.

In summary, our results highlight ERBB4 as a marker of metastatic or relapsed ES. We demonstrated moderate decreases in ERBB4 activity by treatment with Lapatinib, a pan-ERBB inhibitor previously used to target ERBB4 in melanoma (Prickett et al, 2009). This provides preliminary evidence that therapeutic inhibition of ERBB4, such as through new generation ERBB4 inhibitors, may be a tractable strategy for the prevention or treatment of metastatic ES.

## MATERIALS AND METHODS

### Cell lines and antibodies

TC71, TC32, TTC 446 and TC135 ES cells lines were purchased from American Type Culture Collection. SK-N-MC, COG-E-352, ES-5838 and CHLA-25 cell lines were contributed by one of the co-authors, TJT. The human ES cell lines CHLA-9, CHLA-10, CHLA-258 were established as described (Batra et al, 2004). Biological characteristics and culture conditions for each cell line are summarized in Supporting Information Table S2.

### ERBB4 immunoblotting and immunoprecipitation

ERBB4 tyrosine phosphorylation was assessed in CHLA-10 cells grown as M or S, and treated with 1 μM Lapatinib for 24 h. Cells were lysed and immunoprecipitation was performed as previously described (Kang et al, 2007), using an anti-ERBB4 Ab (Santa Cruz Biotechnology). Samples were immunoblotted with anti-phosphotyrosine (p-Tyr) and ERBB4 Abs (Cell Signaling). Detailed descriptions and dilutions of all antibodies used in this study are provided in Supporting Information Table S5.

**The paper explained**

PROBLEM:

Ewing sarcoma (ES) is the second most common bone tumour in children and adolescents. Most ES-related deaths occur due to metastatic disease, and these patients have 5-year survival rates of only 20–25%. Hence, there is an urgent need to identify the biological drivers of metastasis in ES and to develop novel therapeutics that will specifically prevent or target metastases in these patients.

RESULTS:

There are very few reports relating to molecular mediators of metastasis in ES. Here, we show that overexpression of ERBB4, a member of the epidermal growth factor receptor family, is directly linked to metastasis in ES, as inhibition of ERBB4 reduces metastasis formation of ES cells.

IMPACT:Our finding that ERBB4 overexpression is directly linked to *in vivo* metastasis predicts that therapeutic targeting of ERBB4, alone or in combination with cytotoxic agents, could block or target the metastatic potential of ES.

### ERBB4 knockdown experiments

Packaging HEK-293T cells were transfected with p8.91 (gag-pol expressor), pMDG (VSV-G expressor) and a lentiviral construct (either scrambled or two different ERBB4 shRNAs clones) at a ratio of 1:1:1.5 using Opti-MEM (Gibco) and 1 nM polyethyelnimine (PEI; Polysciences). The transfection media was replaced by culture media after 16 h incubation, and viral supernatants were harvested 24, 48 and 72 h later and supplemented with 5 μg/ml polybrene (Sigma-Aldrich) before been filtered and stored at −80°C. ERBB4 shRNAs clones TRCN0000001410 and TRCN0000001411 (ccggcctgtggctattaagattcttctcgagaagaatcttaat agccacaggttttt and ccgggcgcaggaaacatctatattactcgagtaatatagatgtttcctgcgcttttt, respectively) were from Thermo Scientific OpenBiosystems, and were designated 1410 and 1411. A non-silencing-GIPZ lentiviral shRNAmir construct, herein designated Scrambled was used as a control (catalogue RHS4346, Thermo Scientific Openbiosystems). CHLA-9, CHLA-10, CHLA-25, CHLA-258 and SK-N-MC ES cells were transduced with lentiviral particles containing shRNA constructs encoding the ERBB4 shRNA and scrambled sequences and were selected with puromycin-containing media (1 μM; Sigma Aldrich), added every 2 days for 1 week before being plated for experiments.

### Rac1 and Cdc42 Rho GTPase activation assays

Rac1 and Cdc42 GTPase activities were assessed as previously described (Mendoza-Naranjo et al, 2012), in CHLA-9, CHLA-9 cells overexpressing ERBB4, CHLA-10 and ERBB4 shRNA CHLA-10 cells plated for 2 h (cell spreading conditions), or growth as M or S for 24 h. In some experiments, CHLA-10 cells were pre-treated for 16 h with 5 μM UO126 (BD Biosciences), 10 μM LY294002 (BD Biosciences), 1 μM FAK inhibitor II (Calbiochem) or 5 μM FAK inhibitor 14 (Tocris Bioscience) before been plated for 2 h for cell spreading assays, or 24 h under anchorage-independent conditions. Treatments with the inhibitors were kept during those experiments. Rac1 activity was also measured in implantation site tumours isolated from mice implanted with CHLA-10 control or CHLA-10 ERBB4 kd cells (three mice per condition), carefully dissected free of stroma and normal kidney tissue.

### Renal subcapsular implantation (SCI) mouse model

Male, 7-week-old NOD-SCID mice from the Animal Resource Centre BCCRC were maintained according to UBC Animal Care Committee (ACC) regulations. Briefly, cell blocks containing 1 × 10^6^ CHLA-9 pLEOC control, CHLA-9/ERBB4, CHLA-10 Scrambled or CHLA-10 ERBB4 kd ES cell lines were mixed in 50 μl DMEM-rat tail collagen gel, seeded in cell culture plates, incubated for 30 min and then implanted under the capsules of exteriorized kidneys of anaesthetized live NOD-SCID mice; *n* = 8 mice per condition. The kidneys were then gently eased back into the body cavity, the incisions were sutured and the mice were monitored for tumour growth for 7 weeks, in the case of CHLA-10 cells, and 12 weeks for CHLA-9 cells.

### Statistical analysis

Statistical differences were determined using Wilcoxon matched-pairs signed-ranks test for paired data, or analyzed by Bonferroni–Holm post hoc test after one-way analysis of variance (ANOVA) for data sets of multiple comparisons. All data are presented as the mean ± SD except where stated. Criterion levels for the individual tests are given in the Results section. Kaplan–Meier curves and the log-rank test were used to estimate and compare disease-free survival in ES patients. The association between diagnostic clinico-pathological variables and ERBB4 expression was evaluated using the Fisher's exact test. The significance level was set at 5%. Itemized numerical *p*-values and the statistical tests used to generate each *p*-values for all of the experimental conditions analyzed in the manuscript figures are itemized in Supporting Information Table S6.

## Supplementary material

As a service to our authors and readers, this journal provides supporting information supplied by the authors. Such materials are peer reviewed and may be re-organized for online delivery, but are not copy-edited or typeset. Technical support issues arising from supporting information (other than missing files) should be addressed to the authors.
