# Supplementary material for: ERBB4 confers metastatic capacity in Ewing sarcoma
Source: EMBO Mol Med. 2013 May 16;5(7):1019–34. doi: 10.1002/emmm.201202343 (PMC3721475; doi:10.1002/emmm.201202343)
Supplement: Supplementary file 1 [file emmm0005-1019-SD1.pdf]

EMBO Molecular Medicine

ERBB4 confers metastatic capacity in Ewing sarcoma

Ariadna Mendoza-Naranjo, Amal El-Naggar, Daniel H Wai, Priti Mistry, Nikola Lazic, Fernanda Rocha Rojas Ayala, Isabela Werneck da Cunha, Pablo Rodriguez-Viciana, Hongwei Cheng, Jose H Tavares Guerreiro Fregnani, Patrick Reynolds, Robert J. Arceci, Andrew Nicholson, Timothy J Triche, Fernando A. Soares, Adrienne M. Flanagan, Yuzhuo Z Wang, Sandra J. Strauss, Poul H. Sorensen

Corresponding author: Poul Sorensen, British Columbia Cancer Research Centre

Review timeline:

Submission date:	07 December 2012
Editorial Decision:	22 January 2013
Revision received:	09 March 2013
Editorial Decision:	25 March 2013
Revision received:	04 April 2013
Accepted:	05 April 2013

Transaction Report:

(Note: With the exception of the correction of typographical or spelling errors that could be a source of ambiguity, letters and reports are not edited. The original formatting of letters and referee reports may not be reflected in this compilation.)

Editor: Roberto Buccione

1st Editorial Decision 22 January 2013

Thank you for the submission of your manuscript to EMBO Molecular Medicine. We have now heard back from the three Reviewers whom we asked to evaluate your manuscript. You will see that all three Reviewers are supportive of your work and underline its considerable potential interest. They do raise some issues that require your action, however. For this reason, publication of the paper cannot be considered at this stage.

I feel that there is no need to discuss each point in detail as they are clearly stated. Reviewer 1 would like to know whether ERBB4 expression confers increased invasivity without altering growth rates. Furthermore, s/he asks if inhibition of both PI3K and FAK signaling pathways has a synergistic effect on migration and invasion. If such is the case it would imply therapeutical relevance and thus requires direct verification. I agree that it would make an important addition to your manuscript

Reviewer 2 specifically enquires about the p53 status in the patient samples examined and emphasizes the relevance of this for experiments testing the chemosensitivity of ERB4 positive and negative cell lines. Furthermore, Reviewer 2 is concerned that your conclusion that ERBB4 activation in the cell lines is ligand independent is not fully supported by the data, and suggests experimental verification.

Reviewer 3 raises the issue of insufficient experimental support for your contention that the signaling pathway downstream of ERBB4 (which includes AKT and Rac1) supports ERBB4' s ability to promote Ewing sarcoma metastasis. S/he also suggests appropriate experimental strategies

© EMBO

to address this issue.

While publication of the paper cannot be considered at this stage, we would welcome a suitably revised submission, with the understanding that the Reviewers' concerns must be fully addressed with additional experimental data where appropriate.

Please note that it is EMBO Molecular Medicine policy to allow a single round of revision only and that, therefore, acceptance or rejection of the manuscript will depend on the completeness of your responses included in the next, final version of the manuscript.

I look forward to receiving your revised manuscript as soon as possible.

***** Reviewer's comments *****

Referee #1 (Comments on Novelty/Model System):

The experiments have been well designed, performed and analyzed. The authors focused on the metastatic aspect of Ewing sarcoma, when most studies have focused on the EWS-FLI1 translocation product. This makes the study unique and novel. The potential impact of targeting ERBB4 to slow down or prevent metastasis in Ewing sarcoma is very high. The authors use of in vitro and in vivo model systems to address the relevance of ERBB4 in metastasis of Ewing sarcoma is appropriate.

Referee #1 (General Remarks):

This manuscript examines the mechanisms underlying metastasis in Ewing sarcoma using two ES cell lines which differ in their ability to grow in suspension. The findings are then expanded to other ES cell lines. This is a very interesting study looking specifically at metastasis rather than the EWS/FLI1 translocation in ES, and the findings are highly relevant to potential therapy, especially metastatic Ewing sarcoma. The experiments are well designed, the results are consistent and clear, and several independent approaches were taken to reveal the role of ERBB4 in Ewing sarcoma. There are few issues, however, which need to be addressed to further strengthen the manuscript.

Major points:

1. Describe in more detail about the gene expression profiles of CHLA9 vs. CHLA10 cells. For example, how many genes show differential expression and were there genes expressed higher than ERBB4? If so, why were those genes not considered? At least, those genes should be mentioned somewhere in the manuscript.

2. Since S culture condition maximally activates ERBB4 in CHLA10 cells, addition of NRG1/2 or HB-EGF in S condition may not have much of an effect. Also, expression of NRG1 and NRG2 increases dramatically in CHLA10 vs CHLA9 cells grown in M condition, and for NRG2, when CHLA9 cells are cultured in S condition compared to M. These results suggest that activation of ERBB4 and downstream Akt activation may be NRG1- or 2-dependent. In fact, in Supplement Fig. S2G, addition of NRG2 appears to increase pAKT-T308 in CHLA10 cells compared to untreated. Therefore, the statement, "ERBB4 and downstream Akt activation is largely ERBB4-ligand independent" is premature and should be toned down if no further experiments are performed to provide additional evidence. If ERBB4 activation is ligand independent, what is the mechanism in the absence of mutations or gene amplification?

3. Does ERBB4 kd in CHLA10 cells in M condition also cause apoptosis?

4. Does ERBB4 expression confer increased motility (migration/invasion) without altering the growth rate? What are the growth rates of CHLA9 vs CHLA10 cells? Are they comparable? How about the growth rate of CHLA9 cells overexpressing ERBB4 compared to the parental CHLA9 cells?

5. Does inhibition of both PI3K and FAK signaling pathways have any synergistic effects on the migration and invasion? If so, this could be therapeutically relevant and can be tested as in Fig. 8C,

testing each inhibitors alone and in combination, together with etoposide or doxorubicin.

Minor points:

1. TC71 does not show increased ERBB4 protein expression and only a modest increase in RNA when cultured in suspension. This should be mentioned.

2. In Fig. 8A and B, why are CHLA9 (M) cells only surviving at 70%? How is this graph generated?

Referee #2 (Comments on Novelty/Model System):

Disease recurrence and metastasis are the single most-powerful predictors of outcome in Ewing sarcoma. The manuscript provides a novel mechanism of metastasis and identifies a putative therapeutic target. The results are based on cell lines from different disease stages, and a powerful in vivo metastasis model (renal subcapsular implantation mouse model).

Referee #2 (General Remarks):

This paper reports a detailed and very well performed analysis of ERBB4 function in Ewing sarcoma. The authors identify high ERBB4 expression in a subset of Ewing sarcoma cell lines and patient materials, specifically in metastasis derived samples. They provide convincing evidence for ERBB4 being involved in tumor cell migration, invasion, and metastatic spread. They identify PI3K-AKT and FAK activation downstream of ERBB4 under conditions of cellular stress (serum starvation and surface independent growth), which result in activation of Rac GTPase, a well known player in the metastatic process of many cancers. Consistent with their hypothesis that ERBB4 plays a key role in disease progression, the authors report significantly decreased disease-free survival of patients with high ERBB4 expression in their primary tumors. These findings are very important since they may offer a new avenue to the therapeutic targeting of metastatic disease in this aggressive cancer.
As a model to study ERBB4 function, the author used cell lines derived from either pre- (n=3) or post-chemotherapy (n=6) tumor samples. The latter were all mutant for p53 and expressed ERBB4, while the three cell lines established from untreated dignostic biopsies expressed wildtype p53. Mutant p53 has been reported previously to be associated with adverse outcome, and the known biology of mutant p53 allows for many interpretations why it supports treatment resistance. The authors mention that the mechanism of ERBB4 transcriptional activation is unclear. thus, it cannot be excluded that it is related to p53 status. Do the authors know about p53 status in any of the patient samples that they investigated? And, are the authors aware of any ERBB4 negative p53 mutant cell line and, vice versa, any ERBB4 positive cell line that is wildtype for p53? This question becomes even more important in the experiments testing chemosensitivity of ERB4 positive and negative cell lines. Both doxorubicin and etoposide are known to activate the p53 pathway. Thus, it is not surprising that wildtype p53 CHL9 cells were more sensitive than p53 mutant CHL10 cells. It will be difficult to dissect the p53 and the ERBB4 effects in this model system. I therefore suggest to delete the part on chemoresistance from the manuscript.
When referring to the post-chemo cell lines the authors always speak about Ñrecurrent or metastatic ES", however, Table 1 does not provide information on the origin (primary tumor, local relapse or distant metastasis) of these cell lines. The authors should either provide this information if available, or be more acurate by referring simply to Ñpost-chemotherapy" derived cell lines.
The conclusion that ERBB4 activation in the cell lines Ñis largely ligand independent" is not sufficiently supported by the experimental data, which rely solely on the effects of ectopically provided ligands on top of endogenous ligand expression. The authors should perform knockdowns of endogenous ERBB4 ligands HB-EGF, NRG-1, and NRG-2 to make their point. Also, ligand treatment was performed under serum starvation conditions, which the authors show that by itself upregulates ERBB4 activity confounding the experiment.

Minor comments:
Figure S2 C: lacks knockdown-only control
Figure S2 H: lacks legend for bands

Referee #3 (General Remarks):

In the first submission of manuscript EMM-2012-02343 the authors convincingly show that ERBB4 contributes to the metastatic potential of Ewing sarcoma cells and is overexpressed in the metastases of ES patients. In a small cohort, expression of ERBB4 in ES metastasis predicts patient outcome.

This is a thorough and impressive study that addresses an important property of ES metastatic disease. The only additional data that I would recommend is a phenotypic analysis of ERBB4 downstream signaling. The authors put considerable effort into identifying signaling pathways downstream of ERBB4 including AKT and Rac1. They contend that this pathway contributes to ERBB4' s ability to promote ES metastasis, however, this contention needs to be formally tested. Inhibitors are notorious for off target effects. Knockdown experiments of key signaling components downstream of ERBB4 (AKT and Rac) needs to be performed and the impact on CHLA-10 invasion with high basal ERBB4 expression determined.

1st Revision - authors' response 09 March 2013

We would like to thank the reviewers for their comments and suggestions, which have helped to strengthen the manuscript. Point-by-point responses to the reviewers' comments (in italics) are as follow:

Referee #1

Major points:

1. *Describe in more detail about the gene expression profiles of CHLA9 vs. CHLA10 cells. For example, how many genes show differential expression and were there genes expressed higher than ERBB4? If so, why were those genes not considered? At least, those genes should be mentioned somewhere in the manuscript.*

We had previously included a heatmap showing the 46 differentially expressed genes in CHLA-10 versus CHLA-9 ES cells Fig. S1A. In response to the reviewer's comment, we have now added a new table (Supporting Table 1) containing detailed information on the 46 genes from Fig. S1A. This table includes the names of each gene and encoded protein, predicted functions, and known associations with cancer. This is additionally referred to in the revised manuscript on page 5, lines 8-12. Regarding the relative expression of these genes compared to *ERBB4* in CHLA-9 and CHLA-10 cell lines, while Affymetrix expression profiles are very useful to determine differential expression of a given gene in different samples, this method is not reliable for comparing expression changes across different genes. This is because gene-specific probe sets on the chips behave differently and intergene quantitation is unreliable. Therefore we cannot use this data to determine whether other genes are more significantly altered than *ERBB4*. The reason for focusing on *ERBB4* rather than these other genes is that we previously found that ERBB4 is activated in Ewing sarcoma cells under suspension where it acts to suppress *anoikis* (Kang et al, 2007). The latter property is essential for cancer cells to survive under anchorage independent conditions in the circulation or in lymphatics prior to establishment of overt metastases (Simpson et al, Cancer Lett 2008). This point is highlighted in the manuscript on page 5, lines 14-15. We feel that to study all of the differentially regulated genes would be beyond the scope of the current manuscript, and instead we are specifically focusing on one gene of particular interest due to the above reasons.

2. *Since S culture condition maximally activates ERBB4 in CHLA10 cells, addition of NRG1/2 or HB-EGF in S condition may not have much of an effect. Also, expression of NRG1 and NRG2*

increases dramatically in CHLA10 vs CHLA9 cells grown in M condition, and for NRG2, when CHLA9 cells are cultured in S condition compared to M. These results suggest that activation of ERBB4 and downstream Akt activation may be NRG1- or 2-dependent. In fact, in Supplement Fig. S2G, addition of NRG2 appears to increase pAKT-T308 in CHLA10 cells compared to untreated. Therefore, the statement, "ERBB4 and downstream Akt activation is largely ERBB4-ligand independent" is premature and should be toned down if no further experiments are performed to provide additional evidence. If ERBB4 activation is ligand independent, what is the mechanism in the absence of mutations or gene amplification?

We agree that we have not rigorously ruled out a role for ERBB4 ligands based on our findings, especially since ES cells express several known ERBB4 ligands. In addition to the experiment of Fig S2G of the previous version (now Fig. S2H), we also performed another experiment that was previously reported as “data not shown” but is now included as new Supp. Fig S2G. In this experiment we compared effects of ligand addition versus no treatment on ERBB4 tyrosine phosphorylation (Tyr-P) in ES cells, using ERBB4 pulldowns followed by anti-phosphotyrosine immunoblotting. CHLA-9 and CHLA-10 cells were grown as monolayers (M) or under non-adherent conditions (S) and stimulated with *HB-EGF, NRG-1, or NRG-2.* This showed that treatment of CHLA-10 S cultures (but not CHLA-9 S cultures) with exogenous *HB-EGF, NRG-1, or NRG-2 did not significantly increase* ERBB4 Tyr-P (Tyr-P). However, we agree that since ERBB4 appears to be maximally activated under S conditions, and strongly express ERBB4 ligands under these conditions, additional ligand addition may not demonstrably increase Tyr-P. Therefore we have replaced the statement, *"ERBB4 and downstream Akt activation is largely ERBB4-ligand independent"*, with “Our data using ligand stimulation suggest that ERBB4 may already be maximally activated under S conditions, such that additional ectopic ligand treatment is unlikely to further increase ERBB4 P-Tyr and activation. Further experiments are necessary to determine if ERBB4 activation is ligand dependent or independent in ES cells” (page 17, lines 18-22). We also modified the sentence on page, 8, lines 8-9 to reflect this change.

In answer to the question of how ERBB4 might be activated, if indeed ligand independent, since ERBB4 mRNA and protein levels are both increased in CHLA-10 and other chemoresistant ES cell lines, one possibility is that overexpression on the cell surface leads to auto- or cross-activation. In the absence of mutations or gene amplification, the mechanism of transcriptional overexpression remains unknown, although ERBB4 promoter methylation or p53 mediated suppression are possibilities. Since we have now toned down our claim that ERBB4 activation is ligand independent, we have included a sentence addressing the latter points in the Discussion on page 18, lines 1-4.

3. *Does ERBB4 kd in CHLA10 cells in M condition also cause apoptosis?*

We agree that this is an important point. Although we already had performed the relevant experiment, we did not include the data in our previous version as we mainly focused on the effect of ERBB4 knockdown in S cultures. ERBB4 kd also causes apoptosis in CHLA10 cells under M conditions. We have now included this result in the current version as Supp **Fig S2E**, and in the Results section, page 8, lines 1-2.

4. *Does ERBB4 expression confer increased motility (migration/invasion) without altering the growth rate? What are the growth rates of CHLA9 vs CHLA10 cells? Are they comparable? How about the growth rate of CHLA9 cells overexpressing ERBB4 compared to the parental CHLA9 cells?*

We agree that these are important questions. As can seen in the graphs below (Fig. Xi), the growth rates of CHLA-9 (and TC32) parental (pLEOC control) cells versus cells overexpressing ERBB4 are very similar. However, ERBB4 upregulation in these cells do significantly increase migration and invasion (manuscript, Fig 3F and Fig S3E). This strongly suggests that increased proliferative rates

do not explain the observed differences in motility rates.

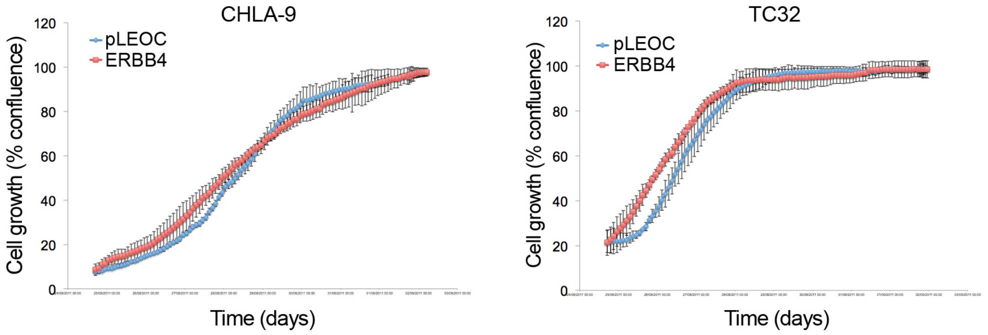

Fig Xi. Growth curves of CHLA-9 and TC32 ES cells transduced with pLEOC vector alone or ERBB4 overexpression constructs are shown. The data represent means ± SD of n=3 experiments.

We did observe significant differences in cell proliferation rates between CHLA-9 and CHLA-10 cells (Fig. Xii graphs below). Although this might suggest a correlation between high proliferative rates and increased migration/invasion in CHLA-10 compared to CHLA-9 cells, there is a large literature indicating that these two phenotypes, proliferation and motility, are not necessarily correlated (e.g. from our group; Evdokimova et al, *Cancer Cell*, 2009; PMID:19411069). Moreover, as shown in Fig Xi, we observed increased motility in CHLA-9 and TC32 cells overexpressing ERBB4 vs. parental cells when there were no differences in growth rates. Therefore we believe that the observed differences in motility are not due to altered proliferation rates.

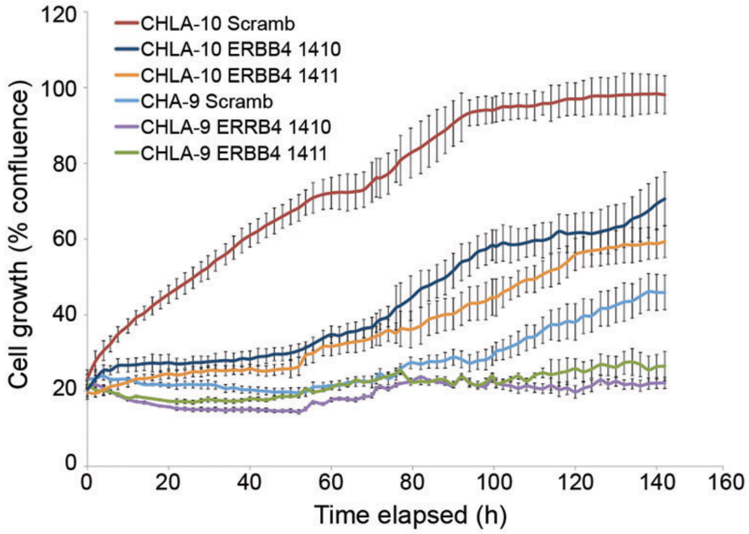

Fig Xii. Incucyte live-cell imaging system was used to generate growth curves of CHLA-9 and CHLA-10 ES cells transduced with scrambled shRNA control versus 1410 or 1411 ERBB4 shRNA constructs as shown. The data represent means ± SD of n=3 experiments.

5. *Does inhibition of both PI3K and FAK signalling pathways have any synergistic effects on the migration and invasion? If so, this could be therapeutically relevant and can be tested as in Fig. 8C, testing each inhibitors alone and in combination, together with etoposide or doxorubicin.*

This is a good suggestion. We combined FAK and PI3K inhibitors and tested whether this has a

© EMBO

synergistic effect on migration and invasion, as suggested by the reviewer (see Fig Xiii below). However, a synergistic effect on invasion and migration was not observed after combining PI3K and FAK inhibitors.

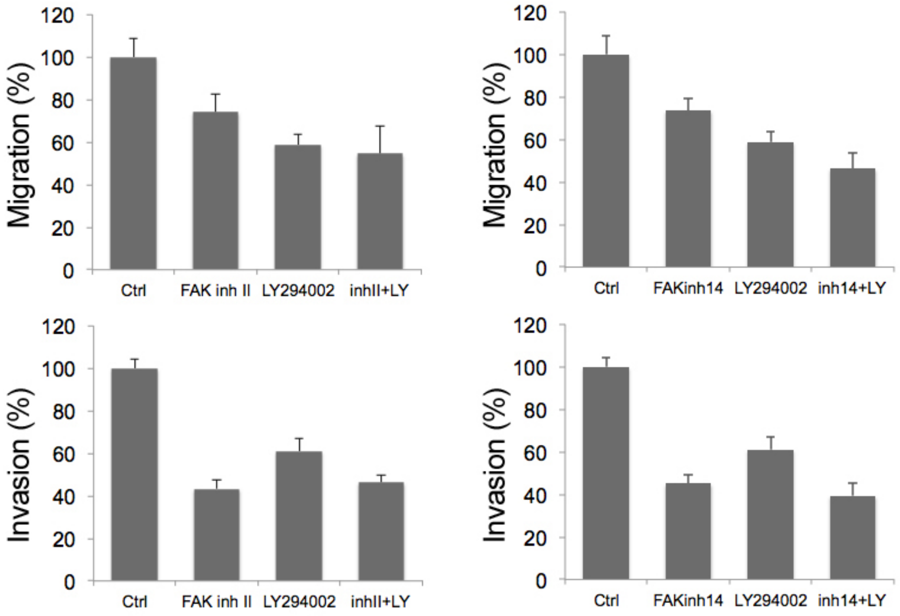

Figure Xiii. Migration and invasion were evaluated using Matrigel BD uncoated or coated Boyden chambers, respectively, in CHLA-10 cells incubated in the presence of 5 μM LY-294002, 5 μM FAKinh14, 1 μM FAKinhII, or combination of LY294002+FAKinhII or LY294002+FAKinh14 added to the lower chambers.

Nevertheless, we still performed the suggested drug combination experiments by combining each of these inhibitors with increasing doses of either Etoposide or Doxorubicin (Fig Xiv, below). No statistically significant differences were observed for any of the combination treatments. Experiments using combinations of FAK and PI3K inhibitors with the above drugs were also performed, but no statistically significant differences were observed after combining these drugs (data not shown). Of note, Reviewer 2, for the reasons outlined below (see Reviewer 2, Point 1), suggests that we delete Fig. 8 relating to drug sensitivity studies of ES cell lines with or without ERBB4 knockdown or Lapatinib treatment from the manuscript. We agree with this comment. Therefore Figs. Xiii and Vix studies have not been included or alluded to in the revised manuscript, as we have removed all experiments related to drug sensitivity testing.

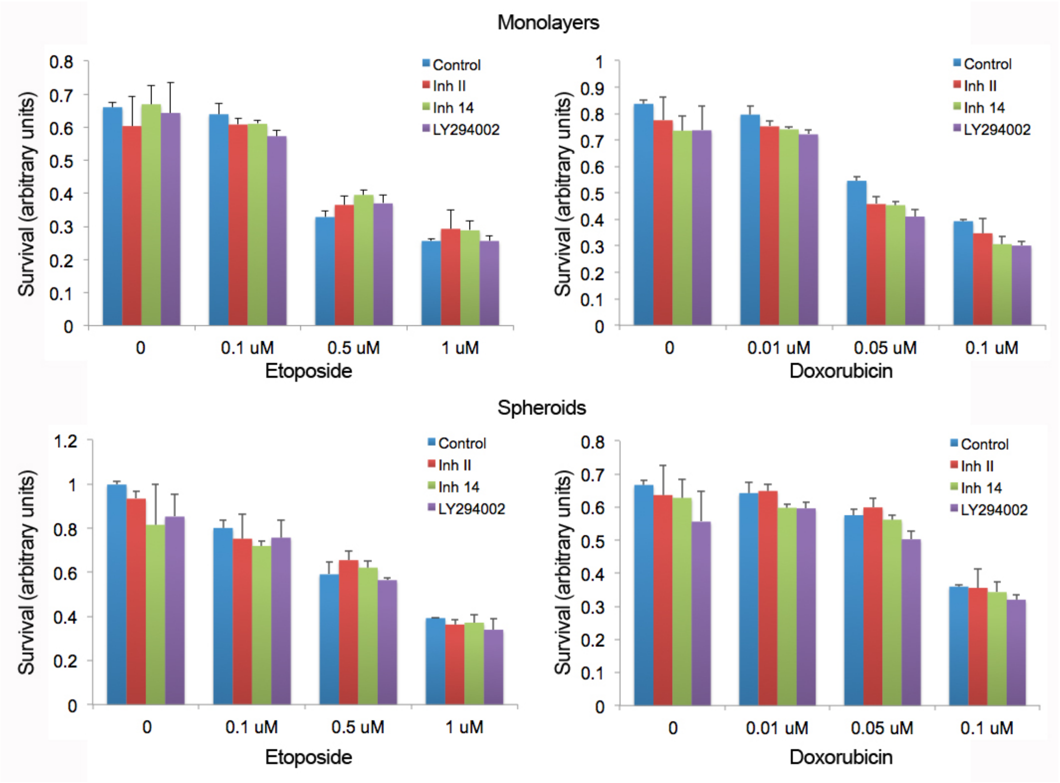

Figure Xiv. Graphs show Etoposide and Doxorubicin dose-response in CHLA-10 cells incubated with 5 μM LY-294002, 5 μM FAKinh14, or 1 μM FAKinhII. Cell viability was measured using the WST-1 assay and was plotted as percentage of cell survival ± SD.

Minor points:

1. *TC71 does not show increased ERBB4 protein expression and only a modest increase in RNA when cultured in suspension. This should be mentioned.*

We have added a sentence stating that only modest increase of ERBB4 expression was seen in TC71 cells in M compared to S cultures. This can be found in page 6, lines 6-7.

2. *In Fig. 8A and B, why are CHLA9 (M) cells only surviving at 70%? How is this graph generated?*

The graph was generated using the maximum value of absorbance in WST-1 assays from 3 separate experiments, which was found in CHLA-10 control cells at 100% survival. The remaining values were calculated with reference to this maximum value. It is conceivable that, since CHLA-9 and CHLA-10 represent different cell lines, they may have different metabolic activities that account for the reduced values observed in CHLA-9 cells. We have now made reference to the aforementioned quantification in the Sup methods section, so this will hopefully be more explanatory for the reader. This can be found in the Supporting Information section, on page 23, lines 13-16

Referee #2

1. The authors mention that the mechanism of ERBB4 transcriptional activation is unclear. thus, it cannot be excluded that it is related to p53 status. Do the authors know about p53 status in any of the patient samples that they investigated? And, are the authors aware of any ERBB4 negative p53 mutant cell line and, vice versa, any ERBB4 positive cell line that is wild type for p53? This question becomes even more important in the experiments testing chemosensitivity of ERB4 positive and negative cell lines. Both doxorubicin and etoposide are known to activate the p53 pathway. Thus, it is not surprising that wild type p53 CHL9 cells were more sensitive than p53 mutant CHL10 cells. It will be difficult to dissect the p53 and the ERBB4 effects in this model system. I therefore suggest to delete the part on chemoresistance from the manuscript.

This is a very relevant set of questions. We do not know the p53 status of any of the Ewing sarcoma clinical samples. Assessing p53 expression in clinical samples by immunohistochemistry (IHC) is often not a reliable indicator of p53 mutation status, as p53 stabilization may occur by other mechanisms. We would therefore have to sequence the samples, but unfortunately we do not have available material for such analyses. Moreover, none of the cell lines used are p53 wild-type and ERBB4 overexpressing, nor the opposite (see Fig. 1E and Supporting Table 2). We therefore agree with the reviewer that it will very difficult to separate out ERBB4 and p53 effects on chemoresistance, and therefore we have deleted all of the drug sensitivity testing experiments (previous Fig. 8) in the revised manuscript.

2. When referring to the post-chemo cell lines the authors always speak about "recurrent or metastatic ES", however, Table 1 does not provide information on the origin (primary tumour, local relapse or distant metastasis) of these cell lines. The authors should either provide this information if available, or be more accurate by referring simply to "post-chemotherapy" derived cell lines.

We agree that for some of the cell lines the origin of the lines is unknown. We have therefore redesignated these cell lines as post-chemotherapy.

3. The conclusion that ERBB4 activation in the cell lines "is largely ligand independent" is not sufficiently supported by the experimental data, which rely solely on the effects of ectopically provided ligands on top of endogenous ligand expression. The authors should perform knockdowns of endogenous ERBB4 ligands HB-EGF, NRG-1, and NRG-2 to make their point. Also, ligand treatment was performed under serum starvation conditions, which the authors show that by itself upregulates ERBB4 activity confounding the experiment.

This point is similar to Reviewer 1, Point 2. As discussed in our response to that point, we agree that since ERBB4 appears to be maximally activated in CHLA-10 cells under S conditions, and that the same cells express high levels of ERBB4 ligands under these conditions, additional ectopic ligand addition may not observably increase Tyr-P under these conditions. Therefore we cannot say that ERBB4 activation is or is not ligand independent. We also agree that since serum starvation is expected to increase ERBB4 activation, the results are further confounded. Therefore we have replaced the statement, *"ERBB4 and downstream Akt activation is largely ERBB4-ligand independent"*, with "Our data using ligand stimulation suggest that ERBB4 may already be maximally activated under S conditions, such that additional ectopic ligand treatment is unlikely to further increase ERBB4 P-Tyr and activation. Further experiments are necessary to determine if ERBB4 activation is ligand dependent or independent in ES cells." These can be found in page 17, lines 18-22.

Minor comments:

Figure S2 C: lacks knockdown-only control

The effects of ERBB4 knockdown vs. scrambled control shRNA is depicted for all three cell lines in Fig 2B and 2C.

Figure S2 H: lacks legend for bands

We thank the reviewer for pointing this out. The missing information has been added to the figure in the revised version.

Referee #3

This is a thorough and impressive study that addresses an important property of ES metastatic disease. The only additional data that I would recommend is a phenotypic analysis of ERBB4 downstream signalling. The authors put considerable effort into identifying signalling pathways downstream of ERBB4 including AKT and Rac1. They contend that this pathway contributes to ERBB4' s ability to promote ES metastasis, however, this contention needs to be formally tested. Inhibitors are notorious for off target effects. Knockdown experiments of key signalling components downstream of ERBB4 (AKT and Rac) need to be performed and the impact on CHLA-10 invasion with high basal ERBB4 expression determined.

We agree with this point. To provide genetic evidence, we therefore performed siRNA knockdown of Akt and Rac1 in CHLA-10 cells with high basal ERBB4 expression, as suggested by the reviewer, and performed migration and invasion experiments on these cells compared to control siRNA cells. Additionally, we expressed commercially available Akt and Rac1 dominant negative (DN) constructs in CHLA-10 cells and tested effects on migration and invasion using the same assays. These new data can be found as Fig 5C, and provide compelling evidence that reducing Akt or Rac1 levels or activity markedly reduces both migration and invasion in CHLA-10 cells.

2nd Editorial Decision 25 March 2013

Thank you for the submission of your revised manuscript to EMBO Molecular Medicine. I am pleased to inform you that we will be able to accept your manuscript pending the following final technical amendments:

1) The description of all reported data that includes statistical testing must state the name of the statistical test used to generate error bars and P values, the number (n) of independent experiments underlying each data point (not replicate measures of one sample), and the actual P value for each test (not merely 'significant' or 'P < 0.05'). Please make sure that this if fully complied with.

3) There is space at the end of each article to list relevant web links for further consultation by our readers ("For more information"). Could you identify some relevant ones and provide such information as well? Some examples are patient associations, relevant databases, OMIM/proteins/genes links, author's websites, etc... (this is not compulsory but might apply in your case)

Please submit your revised manuscript within two weeks. Needless to say, the sooner we receive it

the sooner I will be able to formally accept your manuscript.

I look forward to receiving a new revised version of your manuscript as soon as possible.

***** Reviewer's comments *****

Referee #1 (General Remarks):

The authors have sufficiently addressed all the comments.
