# Supplementary material for: ERBB4 confers metastatic capacity in Ewing sarcoma
Source: EMBO Mol Med. 2013 May 16;5(7):1019–34. doi: 10.1002/emmm.201202343 (PMC3721475; doi:10.1002/emmm.201202343)
Supplement: Supplementary file 2 [file emmm0005-1019-SD2.pdf]

SUPPORTING INFORMATION

ERBB4 confers metastatic capacity in Ewing sarcoma (Mendoza-Naranjo *et al*)

Table of contents:

Supporting Figure S1……………………………………………………………page 2
Supporting Figure S2……………………………………………………………page 3-4
Supporting Figure S3……………………………………………………………page 5-6
Supporting Figure S4……………………………………………………………page 7
Supporting Figure S5……………………………………………………………page 8
Supporting Table S1………..………………………………………………………page 9-13
Supporting Table S2………..………………………………………………………page 14
Supporting Table S3………..………………………………………………………page 15
Supporting Table S4………..………………………………………………………page 16
Supporting Table S5………..………………………………………………………page 17
Supporting Table S6………..………………………………………………………page 18-20
Supporting Materials And Methods……………………………………………page 21-29
Supporting References………..……………………...………………………page 30

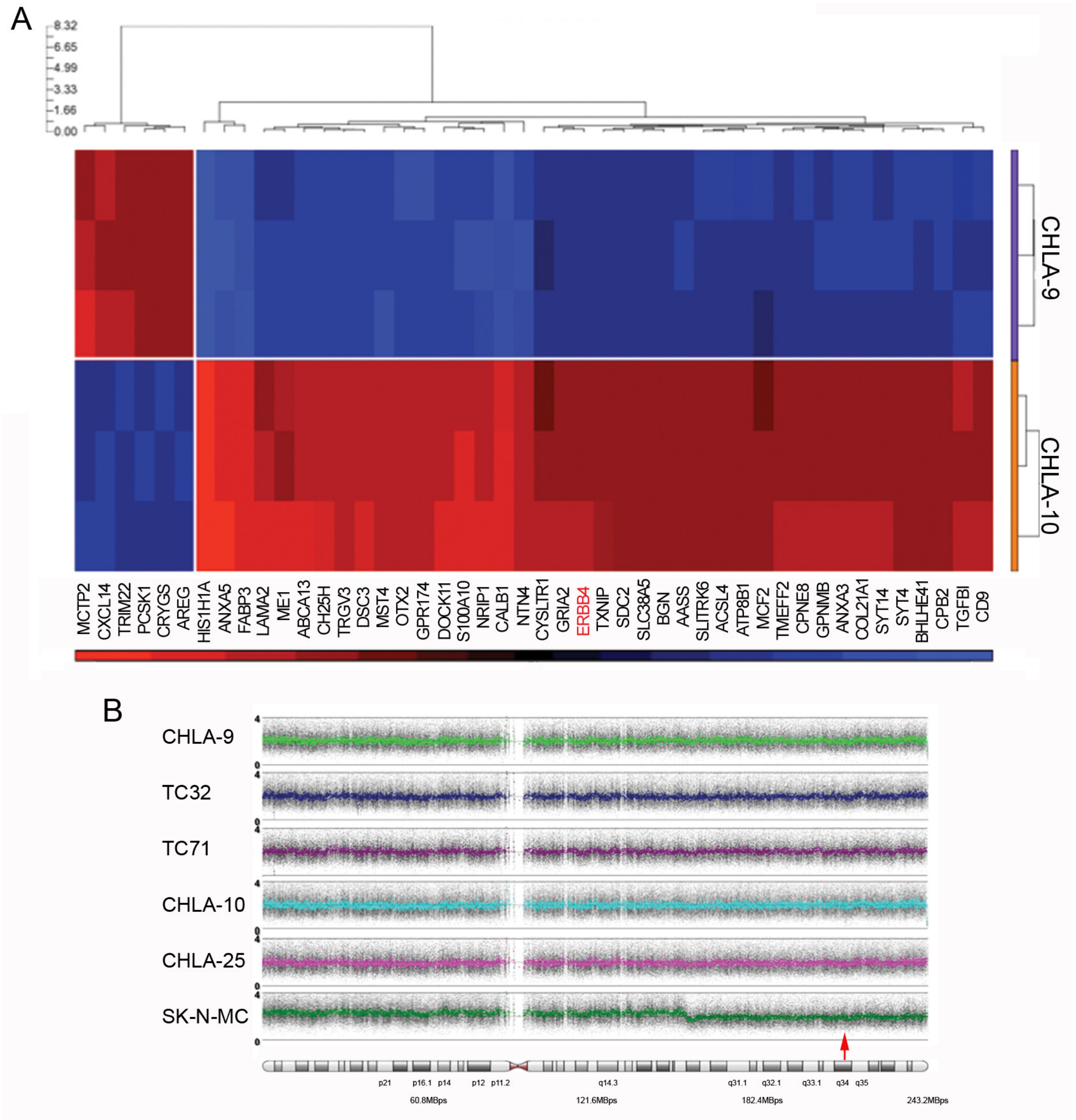

Supporting Figure S1 Regulation of ERBB4 expression in ES cell lines. A. Heat-map showing the expression profiles of 46 genes significantly differentially expressed (at least 7-fold; $p<0.05$, n=3) in CHLA-10 vs. CHLA-9 cells. **B**. Genome-wide copy number analysis of 6 ES cell lines was performed on Affymetrix SNP6.0 arrays. The red arrow indicates the location of *ERBB4* (Chr2q34: 212,240,442 - 213-403,353, hg19). SNP profiles show no obvious copy-number aberrations at the *ERBB4* locus in any of the cell lines analysed.

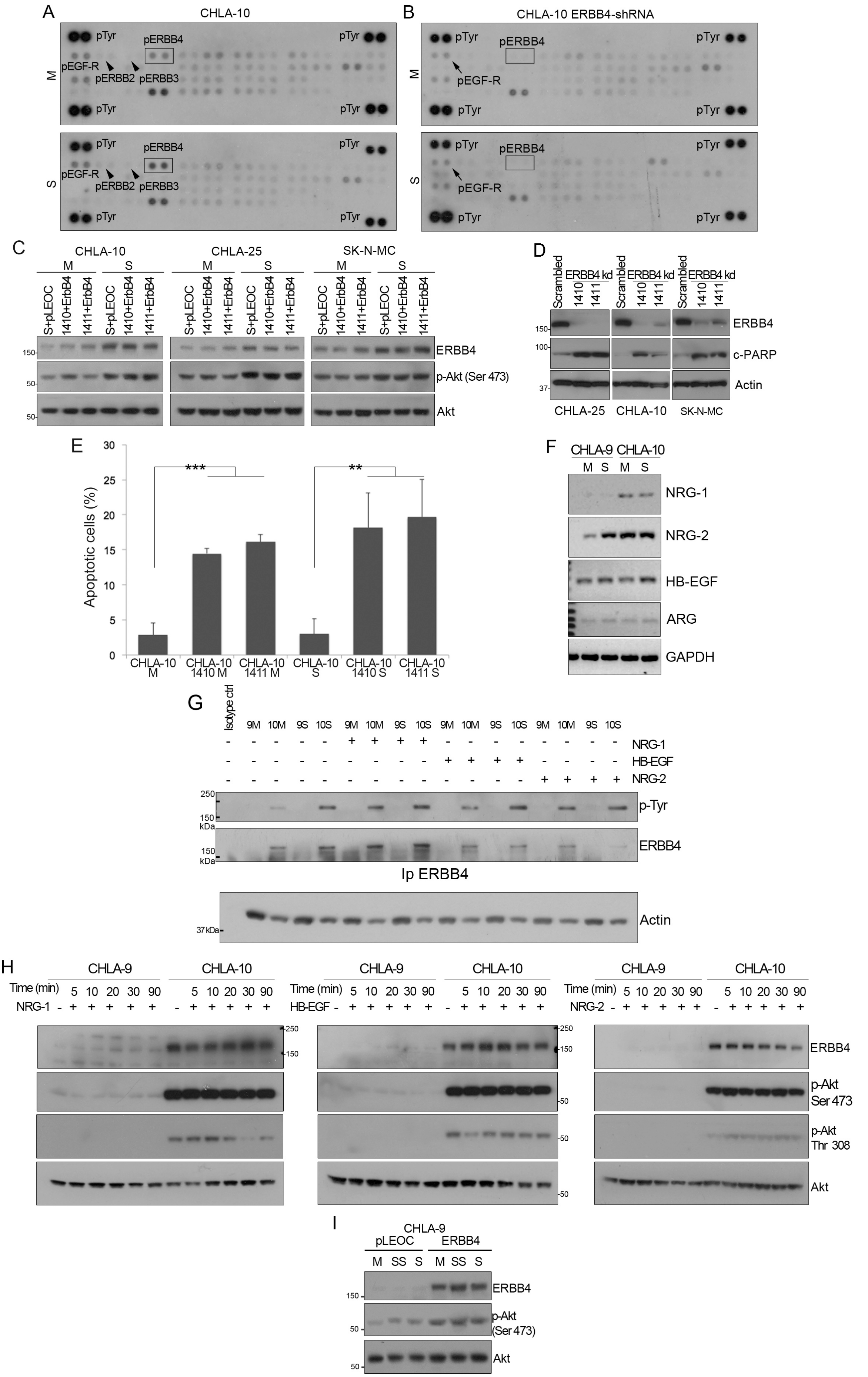
A
CHLA-10
pTyr
pERBB4
pEGF-R
pERBB2
pERBB3
M
S
B
CHLA-10 ERBB4-shRNA
C
CHLA-10
CHLA-25
SK-N-MC
S+pLEOC
1410+ErbB4
1411+ErbB4
ERBB4
p-Akt (Ser 473)
Akt
D
Scrambled
ERBB4 kd
1410
1411
c-PARP
Actin
CHLA-25
SK-N-MC
E
Apoptotic cells (%)
CHLA-10 M
CHLA-10 1410 M
CHLA-10 1411 M
CHLA-10 S
CHLA-10 1410 S
CHLA-10 1411 S
F
CHLA-9
NRG-1
NRG-2
HB-EGF
ARG
GAPDH
G
Isotype ctrl
9M 10M 9S 10S
p-Tyr
Ip ERBB4
H
Time (min)
p-Akt Ser 473
p-Akt Thr 308
I
CHLA-9
pLEOC
M SS S
p-Akt (Ser 473)

Supporting Figure S2 Akt-activity modulation in ES cells and rescue experiments after ERBB4 kd. A-B. The tyrosine kinase activation profiles of 42 tyrosine kinases were evaluated by proteome arrays in CHLA-10 (**A**), and ERBB4 kd CHLA-10 cells (**B**) growth as M or S; n=3. Tyrosine phosphorylation of ERBB4 was evident in these arrays (**A**; black boxes), and was increased under S conditions. Among members of the EGFR family, very weak ERBB2 and ERBB3 p-Tyr was observed, and EGFR p-Tyr levels, although detectable, did not change in M vs. S cultures (**A**, arrows). ERBB4 kd did not affect p-Tyr of any tyrosine kinase analysed (**B**). **C**. Rescue of ERBB4 in 1410 or 1411 ERBB4 kd cells using plasmids containing silent mutations in the ERBB4shRNA target regions restored Akt activation. **D**. ERBB4 kd in 3 metastatic ES cell lines dramatically increased c-PARP expression. Actin was used as a loading control; n=3. **E**. The graph shows apoptosis in CHLA-10 grown as M or S cultures, calculated as described in the Supplementary Methods section. Data represent mean ± SD of three independent experiments; ** $p<0.01$; *** $p<0.005$. Specific numerical *P*-values for these and all subsequent supporting experiments are itemized in Supporting Table S6. **F**. ERBB4 ligands expression was examined by RT-PCR in CHLA-9 and 10 cells cultured as M or S. *GAPDH* was used as an internal control. **G**. ERBB4 tyrosine phosphorylation (p-Tyr) was evaluated after treatment of M or S cultures of CHLA-9 and CHLA-10 cells with the indicated ErbB4 ligands. Cell extracts were immunoprecipitated for endogenous ErbB4, and samples were immunoblotted with P-Tyr or ERBB4 Abs. Total lysates were probed with an Actin antibody as a loading control. **H**. Akt activation was analysed in CHLA-9 and CHLA-10 ES cells incubated with three ERBB4 ligands in the absence of serum. One of three representative experiments is shown. **I**. ERBB4 and p-Akt expression levels were evaluated by Western blot in CHLA-9 cells transduced with ERBB4 or pLEOC constructs. Cells were growth as monolayer in serum-stimulated (M), serum-starved (SS), or under anchorage independent conditions (S) for 24 h. Total Akt was used as a loading control. Blots are representative of n=3 independent experiments.

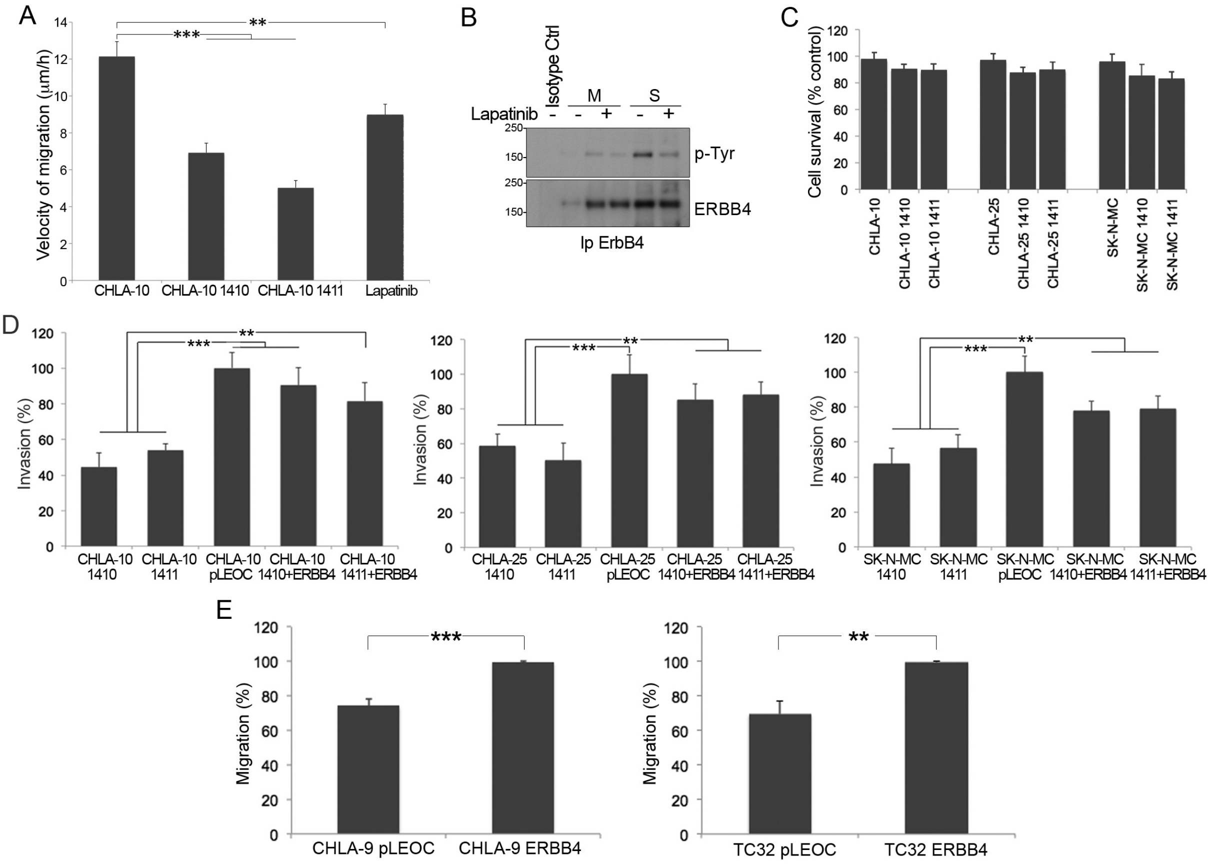

Supporting Figure S3 Targeting ERBB4 impairs migration and invasion of metastatic ES cells. **A**. Confluent monolayers of CHLA-10 cells transduced with 1410 and 1411 ERBB4-shRNA constructs, or treated with 1 µM Lapatinib were wound-scratched and allowed to migrate for 30 h. The graph shows the velocity of migration ± SD of n=6 independent experiments; **, *p*<0.01; ***, *p*<0,005. **B**. The effect of Lapatinib in ERBB4 tyrosine kinase activity was evaluated in CHLA-10 grown as M or S, and treated with 1 µM Lapatinib. Samples were immunoprecipitated with anti-ERBB4 antibody, and immunoblotted with anti-phosphotyrosine (p-Tyr) or ERBB4 Abs. Figure depicts one of three representative experiments. **C**. Cell viability was measured over a period of 24 h using WST-1 assays in Scrambled and ERBB4 kd CHLA-10, CHLA-25 and SK-N-MC cells. The graph represents percent of cell survival ± SD; n=4. **D**. Matrigel-coated transwells were used to analyse the extent of cell invasion in CHLA-10, CHLA-25, and SK-N-MC metastatic cell lines. ERBB4

kd significantly reduced cell invasion through Matrigel-coated transwells; *** $p<0.005$. The extent of cell invasion was reestablished after restoring ERBB4 expression in all three cell lines; ** $p<0.01$. **E**. Boyden chamber experiments were used to analyse the contribution of ERBB4 to the cell migration process in CHLA-9 and TC32 ES ES cells overexpressing ERBB4, or transduced with pLEOC control constructs. Data represent mean ± SD of three independent experiments; **$p<0.01$; ***$p<0.005$.

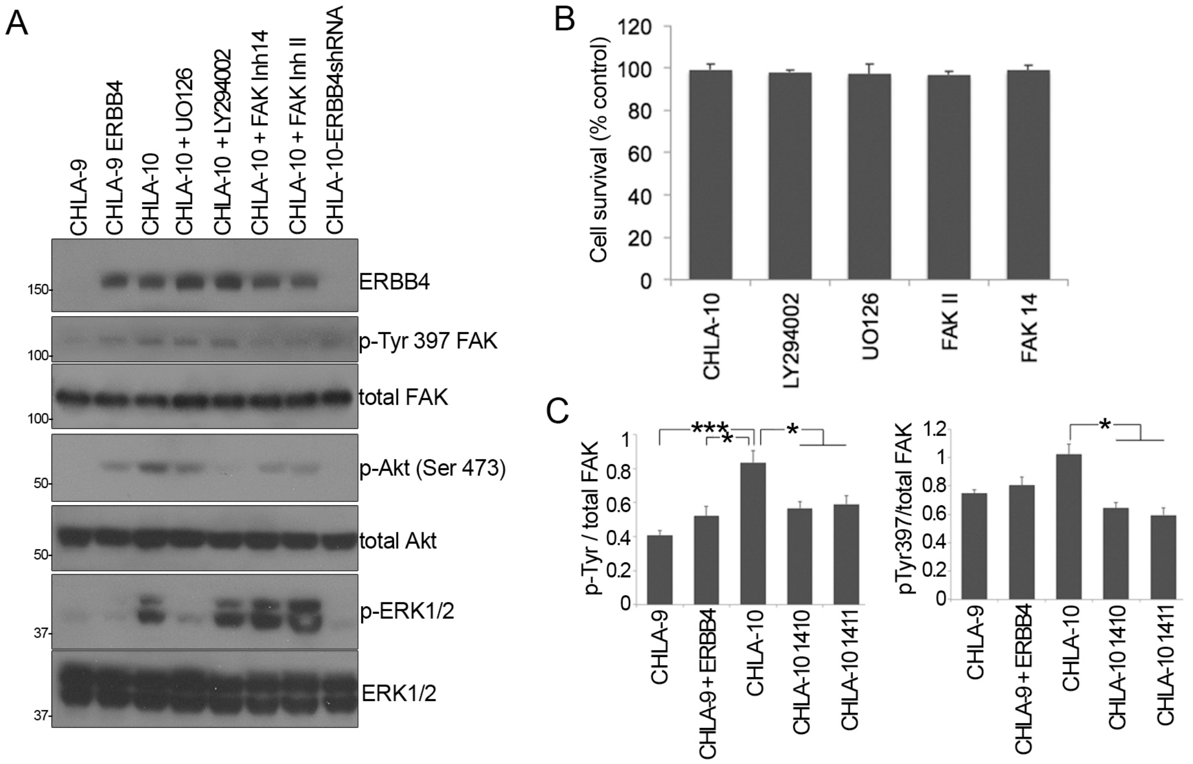

Supporting Figure S4 ERBB4 regulates FAK activity in ES cells. A. CHLA-9 cells (scrambled or overexpressing ERBB4), and CHLA-10 cells treated or not with LY-294002, UO-126 and FAKinh14 or FAKinhII, were harvested, lysed, and processed by Western blot as described in the Methods section. Figure depicts representative blots from n=3 independent experiments. **B**. Cell viability was measured over a period of 40 h using WST-1 assays in CHLA-10 cells treated with vehicle or LY-294002, UO-126, FAKinh14 and FAKinhII. The graph represents percentage of cell survival ± SD. **C**. Total FAK Tyr and Tyr397 phosphorylation were assessed by immunoprecipitation in CHLA-9, CHLA-9-ERBB4, CHLA-10, and CHLA-10 ERBB4 kd (1410 and 1411) cells, during cell spreading assays. Graphs show p-Tyr 397 and total P-Tyr levels normalized vs. total FAK among the different conditions; * $p<0.05$; *** $p < 0.005$; n=3.

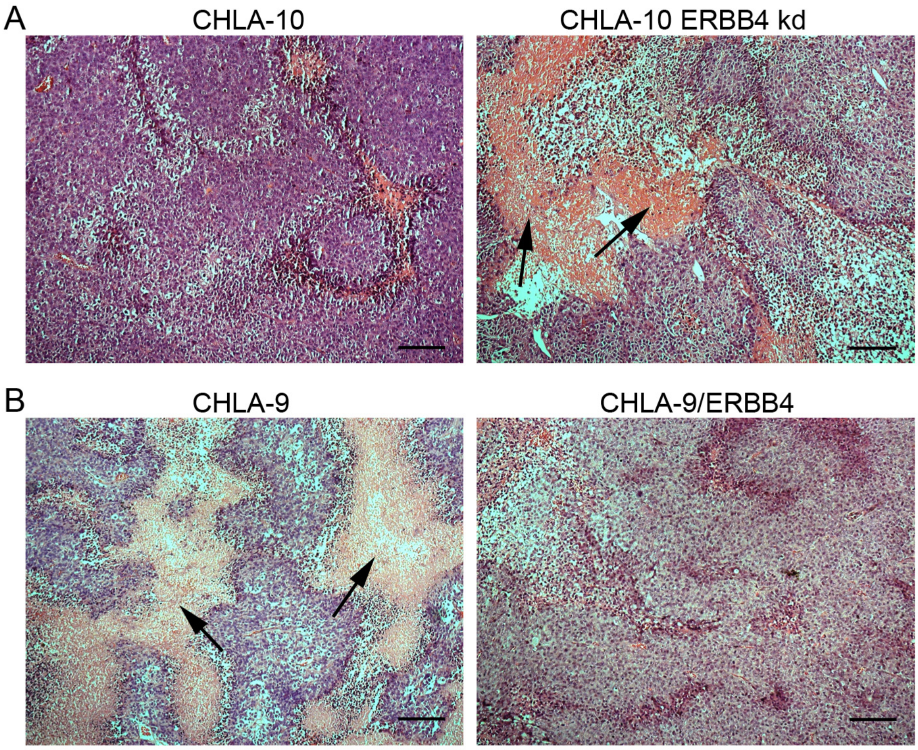

Supporting Figure S5 ERBB4 contributes to invasion and metastasis formation *in vivo*. **A**-**B**. H&E staining showing that CHLA-10 and CHLA-10 ERBB4 kd (**A**), and CHLA-9 and CHLA-9/ERBB4 cells (**B**) formed large tumours under the renal capsule of implanted mice. Scale bars = 50 μm. Arrows denote regions of necrosis in CHLA-10 ERBB4 kd (**A**) and CHLA-9 tumours (**B**).

Supporting Table S1 List of genes significantly differentially expressed (at least 7-fold; $p<0.05$) in CHLA-10 versus CHLA-9 Ewing sarcoma cell lines, along with encoded proteins, and function and relation to cancer if known. Genes shown in red were significantly upregulated whereas those in blue were downregulated.

Gene	Protein	Description/Function	Relation to Cancer
ANXA5	annexin A5	Annexin 5 is a phospholipase A2 and protein kinase C inhibitory protein with calcium channel activity and a potential role in cellular signal transduction, inflammation, growth and differentiation.	Expression of annexin a5 is associated with higher tumor stage and poor prognosis in colorectal adenocarcinomas
FABP3	fatty acid binding protein 3	Fatty acid-binding protein 3 gene contains four exons and its function is to arrest growth of mammary epithelial cells.	This gene is a candidate tumor suppressor gene for human breast cancer.
LAMA2	laminin, alpha 2	This gene encodes the alpha 2 chain, which constitutes one of the subunits of laminin 2 (merosin) and laminin 4 (s-merosin). Mutations in this gene have been identified as the cause of congenital merosin-deficient muscular dystrophy.	LAMA2 is a DNA methylation marker in colorectal cancer
ME1	malic enzyme 1, NADP(+)-dependent, cytosolic	This gene encodes a cytosolic, NADP-dependent enzyme that generates NADPH for fatty acid biosynthesis.	ME1 and ME2 are often overexpressed in cancers and can suppress senescence and promote tumour growth.
ABCA13	ATP-binding cassette, sub-family A (ABC1), member 13	This gene is a member of ABC gene subfamily A (ABCA).	ABCA13 was upregulated in colorectal tumours versus control tissues
CH25H	cholesterol 25-hydroxylase	This is an intronless gene that is involved in cholesterol and lipid metabolism.	Unknown
TRGV3	T cell receptor gamma variable 3	The functional role is still unclear	Possible role in leukaemia
DSC3	desmocollin 3	The protein encoded by this gene is a calcium-dependent glycoprotein and component of intercellular desmosome junctions. Involved in the interaction of plaque proteins and intermediate filaments mediating cell-cell adhesion.	Methylation of DSC3 DNA is a prognostic marker in human colorectal cancer
MST4	Mammalian STE20-like protein kinase 4	The product of this gene is a member of the GCK group III family of kinases. Mediator of cell growth. Modulates apoptosis	MST4 plays a role in prostate cancer progression.
OTX2	orthodenticle homeobox 2	This gene encodes a member of the bicoid subfamily of homeodomain-containing transcription factors. The encoded protein acts as a transcription factor and plays a role in brain, craniofacial, and sensory organ development.	OTX2 is essential in medulloblastoma and directly drives proliferation by regulation of cell cycle genes.
GPR174	G protein-coupled receptor 174	This gene encodes a protein belonging to the G protein-coupled receptor superfamily. Putative receptor for purines coupled to G-proteins	GPR174 was statistically significantly overexpressed in subcutaneous metastases in melanoma
DOCK11	dedicator of cytokinesis 11	Guanine nucleotide-exchange factor (GEF) that activates CDC42 by exchanging bound GDP for free GTP	DOCK11 is amongst the top-20 highest expressed genes in testicular carcinoma

S100A10	S100 calcium binding protein A10	The protein encoded by this gene is a member of the S100 family of proteins and is involved in the regulation of a number of cellular processes such as cell cycle progression and differentiation. S100A10 is a key regulator of cellular plasmin production.	S100A10 plays two major roles in oncogenesis, first as a regulator of cancer cell invasion and metastasis and secondly as a regulator of the recruitment of tumour-associated cells, such as macrophages, to the tumour site.
NRIP1	nuclear receptor interacting protein 1	NRIP1 is a nuclear protein that interacts with the hormone-dependent activation domain AF2 of nuclear receptors. This protein modulates transcriptional activity of the estrogen receptor.	NRIP1 (RIP140) regulates retinoic acid mediated differentiation and growth suppression of human embryonal carcinoma cells and the proliferation of breast cancer cells *in vitro*. A potential role for RIP140 in cancer cachexia has been suggested.
CALB1	calbindin 1	Calbindin is a calcium-binding protein belonging to the troponin C superfamily. Buffers cytosolic calcium. May stimulate a membrane Ca(2+)-ATPase and a 3',5'-cyclic nucleotid phosphodiesterase	Possible role in lung cancer
NTN4	netrin 4	NTN4 belongs to a family of proteins related to laminins. May play an important role in neural, kidney and vascular development.	NTN4 promotes glioblastoma cell proliferation; is upregulated in breast carcinoma effusions compared to corresponding solid tumours
CYSLTR 1	cysteinyl leukotriene receptor 1	Receptor for cysteinyl leukotrienes mediating bronchoconstriction of individuals with and without asthma. Stimulation by LTD4 results in the contraction and proliferation of smooth muscle, edema, eosinophil migration and damage to the mucus layer in the lung.	Possible role in gastric carcinogenesis
GRIA2	glutamate receptor, ionotropic, AMPA 2	Receptor for glutamate that functions as ligand-gated ion channel in the central nervous system and plays an important role in excitatory synaptic transmission. L-glutamate acts as an excitatory neurotransmitter at many synapses in the central nervous system.	Expression of GRIA2 predicts better survival in advanced ovarian adenocarcinomas
ERBB4	Tyrosine kinase-type cell surface receptor HER4	Tyrosine-protein kinase that plays an essential role as cell surface receptor for neuregulins and EGF family members and regulates development of the heart, the central nervous system and the mammary gland, gene transcription, cell proliferation, differentiation, migration and apoptosis.	ERBB4 activation is linked to anoikis suppression in Ewing sarcoma. Mutationally activated ERBB4 alleles were in 20% of melanoma cases. ERBB4 is an independent prognostic factor in serous ovarian cancer and selectively promotes ovarian cancer cell growth in vitro. Nuclear ERBB4 associated with inferior survival of breast cancer patients as compared with membranous staining.
TXNIP	thioredoxin interacting protein	May act as an oxidative stress mediator by inhibiting thioredoxin activity or by limiting its bioavailability. Required for the maturation of natural killer cells. Acts as a suppressor of tumour cell growth	It has been identified as a tumour suppressor gene (TSG) in various solid tumours and haematological malignancies.
SDC2	Syndecan 2	The protein encoded by this gene is a transmembrane (type I) heparan sulfate proteoglycan and is a member of the syndecan proteoglycan family.	Altered syndecan-2 expression has been detected in several different tumour types.
SLC38A 5	solute carrier family 38, member 5	Functions as a sodium-dependent amino acid transporter which countertransport protons. Mediates the saturable, pH-sensitive, and electrogenic cotransport of several neutral amino acids including glycine, asparagine, alanine, serine, glutamine and histidine with sodium	Unknown

BGN	biglycan	The protein encoded by this gene is a small cellular or pericellular matrix proteoglycan that is closely related in structure to two other small proteoglycans, decorin and fibromodulin. This protein plays a role in assembly of collagen fibrils and muscle regeneration.	Biglycan is a specific marker and an autocrine angiogenic factor of tumour endothelial cells.Biglycan expression correlates with aggressiveness and poor prognosis of gastric cancer. Up-regulated biglycan expression correlates with the malignancy in human colorectal cancers. Biglycan is overexpressed in pancreatic cancer
AASS	aminoadipate-semialdehyde synthase	Bifunctional enzyme that catalyzes the first two steps in lysine degradation.	Unknown
SLITRK6	SLIT and NTRK-like family, member 6	SLITRK6 is an integral membrane protein with 2 N-terminal leucine-rich repeat (LRR) domains. Suppresses neurite outgrowth	DNA loss was identified in 16% of cases in a Trk-T1 transgenic mouse model of thyroid neoplasia
ACSL4	acyl-CoA synthetase long-chain family member 4	The protein encoded by this gene is an isozyme of the long-chain fatty-acid-coenzyme A ligase family that converts free long-chain fatty acids into fatty acyl-CoA esters, and thereby play a key role in lipid biosynthesis and fatty acid degradation.	ACSL4 is increased in breast, colon and hepatocellular carcinoma.
ATP8B1	ATPase, aminophospholipid transporter, class I, type 8B, member 1	This gene encodes a member of the P-type cation transport ATPase family, which belongs to the subfamily of aminophospholipid-transporting ATPases. May play a role in the transport of aminophospholipids from the outer to the inner leaflet of various membranes and the maintenance of asymmetric distribution of phospholipids in the canicular membrane.	Polymorphisms in ATP8B1 could be possible risk modifiers in cholangiocarcinoma
MCF2	MCF.2 cell line derived transforming sequence	Guanine nucleotide exchange factor (GEF) that modulates the Rho family of GTPases. Promotes the conversion of some member of the Rho family GTPase from the GDP-bound to the GTP-bound form.	The proto-Dbl (MCF2) transcript was detected at high frequency in some tumoral histio types of neuroectodermal and neuroendocrine origin.
TMEFF2	transmembrane protein with EGF-like and two follistatin-like domains 2	May be a survival factor for hippocampal and mesencephalic neurons.	The shedded form up-regulates cancer cell proliferation, probably by promoting ERK1/2 phosphorylation. Methylation-associated down-regulation of TMEFF2 gene may be involved in lung tumorigenesis and TMEFF2 methylation can serve as a specific blood-based biomarker for NSCLC.
CPNE8	copine VIII	May function in membrane trafficking. Exhibits calcium-dependent phospholipid binding properties	Unknown
GPNMB	glycoprotein (transmembrane) nmb	The protein encoded by this gene is a type I transmembrane glycoprotein which shows homology to the pMEL17 precursor, a melanocyte-specific protein.	GPNMB is highly expressed in metastatic melanoma and in other tumours
ANXA3	annexin A3	This protein functions in the inhibition of phopholipase A2 and cleavage of inositol 1,2-cyclic phosphate to form inositol 1-phosphate. This protein may also play a role in anti-coagulation.	Up-regulation of Anxa3 is found to be correlated with enhanced drug resistance of ovarian cancer, to promote the developments of colorectal adenocarcinoma and pancreatic carcinoma, and to facilitate the metastases of lung adenocarcinoma and hepatocarcinoma

COL21A 1	collagen, type XXI, alpha 1	This gene encodes the alpha chain of type XXI collagen, a member of the FACIT collagen family (fibril-associated collagens with interrupted helices). Type XXI collagen is localized to tissues containing type I collagen so, like other members of this collagen family, it may serve to maintain the integrity of the extracellular matrix.	Unknown
SYT14	synaptotagmin XIV	This gene is a member of the synaptotagmin gene family and encodes a protein similar to other family members that mediate membrane trafficking in synaptic transmission. The encoded protein is a calcium-independent synaptotagmin.	Unknown
SYT4	synaptotagmin IV	May be involved in Ca(2+)-dependent exocytosis of secretory vesicles through Ca(2+) and phospholipid binding to the C2 domain or may serve as Ca(2+) sensors in the process of vesicular trafficking and exocytosis	Unknown
BHLHE4 1	basic helix-loop-helix family, member e41	The encoded protein functions as a transcriptional repressor and as a regulator of molecular clock. Defects in this gene are associated with the short sleep phenotype.	DEC2 (BHLHE41) was up regulated in paclitaxel-treated breast cancer cells and has anti-apoptotic effects on the paclitaxel-induced apoptosis in human breast cancer cells. DEC2 regulates pro-apoptotic factor Bim in human oral cancer cells.
CPB2	carboxypeptidas e B2 (plasma)	The protein encoded by this gene is activated by trypsin and acts on carboxypeptidase B substrates. Cleaves C-terminal arginine or lysine residues from biologically active peptides and down-regulates fibrinolysis.	CPB2 cleaves osteoporin that contributes to malignant glioblastoma development
TGFB1	transforming growth factor, beta 1	Multifunctional protein that controls proliferation, differentiation and other functions in many cell types.	This gene is frequently upregulated in tumour cells. Expression of TGF-β1 was significantly associated with aggressive behaviour and shorter disease specific survival in non-gastrointestinal stromal tumour soft tissue sarcomas.
CD9	CD9 molecule	This gene encodes a member of the transmembrane 4 superfamily, also known as the tetraspanin family. Involved in platelet activation and aggregation. Regulates paranodal junction formation. Involved in cell adhesion, cell motility and tumour metastasis.	Overexpression of CD9 in human breast cancer cells promotes the development of bone metastases. CD9 is a potent prognostic marker in gastric gastrointestinal stromal tumours. CD9 mediates chemoresistance in small cell lung cancer.
HISIH1A	histone cluster 1, H1a	This gene is intronless and encodes a member of the histone H1 family. This gene is found in the large histone gene cluster on chromosome 6.	Unknown
MCTP2	multiple C2 domains, transmembrane 2	Belongs to the MCTP family. Five isoforms of the human protein are produced by alternative splicing. MCTP2 contains C2 domains, which bind calcium in the absence of phospholipids.	Unknown
CXCL4 (or PF4)	platelet factor 4	This gene encodes a member of the CXC chemokine family. This chemokine is released from the alpha granules of activated platelets in the form of a homotetramer which has high affinity for heparin and is involved in platelet aggregation. This protein is chemotactic for numerous other cell type and also functions as an inhibitor of hematopoiesis, angiogenesis and T-cell function.	PF4 transfection into tumour cells inhibits angiogenesis, tumour growth and metastasis, and inhibits myeloma proliferation and angiogenesis *in vivo*.

TRIM22	tripartite motif containing 22	The protein encoded by this gene is a member of the tripartite motif (TRIM) family. The TRIM motif includes three zinc-binding domains, a RING, a B-box type 1 and a B-box type 2, and a coiled-coil region. This protein localizes to the cytoplasm and its expression is induced by interferon.	TRIM22 could be involved in proliferation and/or differentiation of leukemic cells.
PCSK1	proprotein convertase subtilisin/kexin type 1	The encoded protein is a type I proinsulin-processing enzyme that plays a key role in regulating insulin biosynthesis.	This encoded protein is associated with carcinoid tumors.
AREG	amphiregulin	Amphiregulin (AREG) is one of the ligands of the epidermal growth factor receptor (EGFR). AREG plays a central role in mammary gland development and branching morphogenesis in organs and is expressed both in physiological and in cancerous tissues.	AREG has functional role in several aspects of tumorigenesis, including self-sufficiency in generating growth signals, limitless replicative potential, tissue invasion and metastasis, angiogenesis, and resistance to apoptosis.
CRYGS	crystallin, gamma S	This gene encodes a protein initially considered to be a beta-crystallin but the encoded protein is monomeric and has greater sequence similarity to other gamma-crystallins. This gene encodes the most significant gamma-crystallin in adult eye lens tissue.	Unknown

Supporting Table S2 Pathological features and culture conditions of cell lines used in this study

Cell line	Location	Source	p53 status	Chemotherapy prior to cell line established	Growth Conditions
CHLA-9	Thoracic	Established at diagnosis	F	None	20% FBS- IMDM+ 1% ITS
TC32	Pelvic	Established at diagnosis	F	None	10% FBS-RPMI
TC135	Thigh, quadriceps mass	Established at diagnosis	F	None	10% FBS-RPMI
TC71	Humerus	Post-chemo	N	Biopsy of locally recurrent tumour (originally metastatic)	10% FBS-RPMI
CHLA-10	Thoracic	Post-chemo	N	Cisplatin, etoposide, doxorubicin, cyclophosphamide	20% FBS- IMDM+ 1% ITS
CHLA-258	Lung metastasis	Post-chemo	N	Post-myeloablative chemotherapy	10% FBS- IMDM
CHLA-25	Unknown	Post-chemo	N	etoposide, ifosfamide, MESNA, vincristine, cyclophosphamide	10% FBS- IMDM
COG-E-352	Fibula	Post-chemo	N	Vincristine/adriamycin /cyclophosphamide, alternating with ifosfamide/etoposide, followed by high dose carboplatin/ifosfamide etoposide	10% FBS-RPMI
SK-N-MC	Retroorbital metastasis	Post-chemo	N	Vincristine, Cyclophosphamide, Doxorubicin, Actinomycin	10% FBS-RPMI
HEK-293T	-	Human embryonic kidney	N	Human embryonic kidney	10% FBS-DMEM

Table S2 legend

For p53 status; F: Functional; N: Non-functional

ITS: Insulin transferrin selenium media supplement (Invitrogen)

IMDM: Iscove's Modified Dulbecco's Medium (Invitrogen)

FBS: Fetal bovine serum (Invitrogen)

RPMI media (Invitrogen)

Supporting Table S3 Characteristics of a cohort of nineteen patients with primary and metastatic samples.

**Characteristics of 19 ES patients with paired primary and metastatic samples**	
Median age, years (range)	19 (12-39)
**Lung metastases (n=8)**	
Median age, years (range)	17 (12-39)
Primary site	
Extremity	4
Non-extremity	4
Time from diagnosis to lung biopsy /resection, median (range)	39 (7-61)
Median overall survival, months (range)	NR (3-36)
**Bone Marrow metastases (n=9)**	
Median age, years (range)	21 (15-26)
Primary site	
Extremity	3
Non-extremity	6
Time of bone marrow biopsy	At diagnosis
Median overall survival, months (range)	15 (7-51)
**Bone metastases (n=2)**	
Median age, years	12 (7-16)
Primary site	
Extremity	1
Non-extremity	1
Time from diagnosis to biopsy of bone metastasis, median (range)	4 (0-8)
Median overall survival, months (range)	12 (11-18)

Supporting Table S4 Clinicopathological characteristics of 48 patients with low versus high ERBB4 expression

ERBB4 expression in ES		Absent /low expression	Moderate/high expression	*P* value (#)
Total number of patients		22	26	
Gender				0.555
	Male	15	15	
	Female	7	11	
Ethnicity				0.687
	Caucasian	18	23	
	Other	4	3	
Age				0.259
	<15 years	14	12	
	>15 years	8	14	
Metastases at diagnosis				0.543
	No	14	19	
	Yes	8	7	
Primary tumour site				0.393
	Extremity	14	13	
	Non-extremity	8	13	
Primary tumour				0.106
	Bone	21	20	
	Soft tissue	1	6	

Supporting Table S5 Description, origin and dilutions of all antibodies used in this study.

Antibodies	Company/catalog number	Dilution
ERBB4	Cell Signaling, 4795	1:750
ERBB4	Santa Cruz, sc-283	1:1000
Phospho Tyrosine	Santa Cruz, sc-508	1:1000
Total ERK1/2	Cell Signaling, 9102	1:1000
Phospho ERK1/2	Cell Signaling, 4370	1:1000
Total Akt	Cell Signaling, 9272	1:2000
Phospho Akt Ser473	Cell Signaling, 9271	1:2000
Phospho Akt Thr308	Cell Signaling, 9275	1:1000
Rac1	BD Transduction Laboratories, 610650	1:2000
Cdc42	BD Transduction Laboratories, 610927	1:1000
FAK	Santa Cruz, sc-558	1:1000
Phospho FAK Tyr397	Cell Signaling, 3283	1:1000
Actin	Sigma, A5316	1:5000
c-PARP Asp214	Cell Signaling, 5625	1:1000

Supporting Table S6 Numerical *P* values and specific statistical tests used in article figures.

Figure	Conditions		*P* value	Statistical test used
**1C** (ERBB4 Jma)	CHLA-9M	CHLA-10M	1.9754E-05	ANOVA (Bonferroni-Holm post hoc)
	CHLA-9M	CHLA-10S	0.000597972	
	CHLA-9S	CHLA-10M	0.000833612	
	CHLA-9S	CHLA-10S	0.001029301	
**1C** (ERBB4 CYT1)	CHLA-9M	CHLA-10M	0.00018404	ANOVA (Bonferroni-Holm post hoc)
	CHLA-9M	CHLA-10S	0.001148124	
	CHLA-9S	CHLA-10M	0.017569573	
	CHLA-9S	CHLA-10S	0.00378169	
**1C** (ERBB4 CYT2)	CHLA-9M	CHLA-10M	0.008151438	ANOVA (Bonferroni-Holm post hoc)
	CHLA-9M	CHLA-10S	0.003036915	
	CHLA-9S	CHLA-10M	0.044274017	
	CHLA-9S	CHLA-10S	0.04143885	
**3B**	CHLA-9	CHLA-10	3.07171E-17	ANOVA (Bonferroni-Holm post hoc)
	CHLA-10	CHLA-10 1410	3.89726E-13	
	CHLA-10	CHLA-10 1411	8.9894E-15	
**3C**	CHLA-9 Scramb	CHLA-10 Scramb	4.26309E-13	ANOVA (Bonferroni-Holm post hoc)
	CHLA-10 Scramb	CHLA-10 1410	1.84496E-08	
	CHLA-10 Scramb	CHLA-10 1411	7.90466E-09	
	CHLA-9 Scramb	CHLA-10 1410	1.67289E-08	
	CHLA-9 Scramb	CHLA-10 1411	4.13507E-13	
**3D**	CHLA-25 Scramb	CHLA-25 1410	0.003654356	ANOVA (Bonferroni-Holm post hoc)
	CHLA-25 Scramb	CHLA-25 1411	0.004660768	
**3E**	SK-N-MC Scramb	SK-N-MC 1410	0.001374238	ANOVA (Bonferroni-Holm post hoc)
	SK-N-MC Scramb	SK-N-MC 1411	0.00462441	
**3F**	TC32 pLEOC	TC32 ERBB4	0.02661	Wilcoxon Matched-Pairs Signed-Ranks
	CHLA-9 pLEOC	CHLA-9 ERBB4	0.00275	
**4A**	CHLA-9 Scramb M	CHLA-10 Scramb S	0.017126224	ANOVA (Bonferroni-Holm post hoc)
	CHLA-10 Scramb M	CHLA-10 Scramb S	0.040223007	
	CHLA-10 Scramb S	CHLA-10 1410 S	0.002460646	
	CHLA-10 Scramb S	CHLA-10 1411 S	0.0025	
**4C**	CHLA-9 FS	CHLA-10 FS	4.04774E-17	ANOVA (Bonferroni-Holm post hoc)
	CHLA-9 R	CHLA-10 R	1.37783E-19	
	CHLA-10 FS	CHLA-10 1410 FS	3.86717E-16	
	CHLA-10 FS	CHLA-10 1411 FS	8.98964E-15	
	CHLA-10 R	CHLA-10 1410 R	1.40025E-15	
	CHLA-10 R	CHLA-10 1411 R	6.01641E-11	
**4D**	CHLA-9 Scramb	CHLA-10 Scramb	0.017727245	ANOVA (Bonferroni-Holm post hoc)
	CHLA-10 Scramb	CHLA-10 1410	0.026039803	
	CHLA-10 Scramb	CHLA-10 1411	0.011238132	

**4E**	CHLA-9 Veh	CHLA-9 ERBB4	0.041040926	ANOVA (Bonferroni-Holm post hoc)
	CHLA-9 Veh	CHLA-10 Veh	2.47925E-06	
	CHLA-9 Veh	CHLA-10 UO126	9.29181E-05	
	CHLA-9 Veh	CHLA-10 LY294002	2.42809E-06	
	CHLA-9 Veh	CHLA-10 FAKinh14	5.21638E-06	
	CHLA-9 ERBB4	CHLA-10 Veh	6.27863E-05	
	CHLA-9 ERBB4	CHLA-10 UO126	0.000533042	
	CHLA-9 ERBB4	CHLA-10 LY294002	9.2794E-05	
	CHLA-10 Veh	CHLA-10 LY294002	0.044232869	
	CHLA-10 Veh	CHLA-10 FAKinh14	0.04514013	
	CHLA-10 Veh	CHLA-10 FAKinhII	0.048193482	
**4F**	CHLA-9 Veh	CHLA-9 ERBB4	0.002753734	ANOVA (Bonferroni-Holm post hoc)
	CHLA-10 Veh	CHLA-10 LY294002	0.043257732	
	CHLA-10 Veh	CHLA-10 FAKinh14	0.040851498	
	CHLA-10 Veh	CHLA-10 FAKinhII	0.044684588	
	CHLA-9 Veh	CHLA-10 Veh	0.001228594	
	CHLA-9 ERBB4	CHLA-10 Veh	0.004780743	
	CHLA-9 Veh	CHLA-10 UO126	0.00469077	
	CHLA-9 ERBB4	CHLA-10 UO126	0.038209269	
	CHLA-9 Veh	CHLA-10 LY294002	0.021589258	
	CHLA-9 Veh	CHLA-10 FAKinh14	0.000450516	
	CHLA-9 Veh	CHLA-10 FAKinhII	0.000707759	
	CHLA-9 ERBB4	CHLA-10 LY294002	0.028708054	
	CHLA-9 ERBB4	CHLA-10 FAKinh14	0.02661532	
	CHLA-9 ERBB4	CHLA-10 FAKinhII	0.02408663	
**5A**	Control	LY294002	5.752E-05	ANOVA (Bonferroni-Holm post hoc)
	Control	FAKinh14	0.017224058	
	Control	FAKinhII	0.018240602	
**5B**	Control	LY294002	0.003776546	ANOVA (Bonferroni-Holm post hoc)
	Control	FAKinh14	3.47639E-10	
	Control	FAKinhII	1.22651E-11	
**5C (migration)**	Scramb	Rac1 kd	3.11664E-07	Wilcoxon Matched-Pairs Signed-Ranks
	Ctrol	Rac1 DN	5.3178E-08	
	Scramb	Akt kd	8.10351E-08	
	Ctrol	Akt DN	0.005814176	
**5C (invasion)**	Scramb	Rac1 kd	6.93326E-08	Wilcoxon Matched-Pairs Signed-Ranks
	Ctrol	Rac1 DN	1.13887E-07	
	Scramb	Akt kd	0.000212785	
	Ctrol	Akt DN	3.75587E-07	
**6B**	CHLA-10	CHLA-10 ERBB4 kd	0.0435	Two samples Z test for proportions (one-tailed)
**6F**	CHLA-9	CHLA-9/ERBB4	0.0476	Two samples Z test for proportions (one-tailed)
**6H**	CHLA-9	CHLA-9/ERBB4	0.0394	Two samples Z test for proportions (two-tailed)
**7C**	CHLA-10	CHLA-10 ERBB4 kd	0.026578546	Wilcoxon Matched-Pairs Signed-Ranks
**7D**	CHLA-10	CHLA-10 ERBB4 kd	0.020648647	Wilcoxon Matched-Pairs Signed-Ranks

**8A**	Primary	Metastasis	0.04323	Fisher's exact test (two sided)
**8B**	High ERBB4 Metastasis	High ERBB4 Primary	5.00E-07	Fisher's exact test (two sided)
	Low ERBB4 Metastasis	Low ERBB4 Primary	5.00E-07	Fisher's exact test (two sided)
**8E**	Absent/weak ERBB4	Strong/moderate ERBB4	0.0132	Gehan-Breslow-Wilcoxon test
**S2E**	CHLA10 M	CHLA10 1410M	0.001238824	ANOVA (Bonferroni-Holm post hoc)
	CHLA10 M	CHLA-10 1411M	0.000471173	
	CHLA-10 S	CHLA-10 1410 S	0.009983927	
	CHLA-10 S	CHLA-10 1411 S	0.007526607	
**S3A**	CHLA-10	CHLA-10 1410	3.89726E-13	ANOVA (Bonferroni-Holm post hoc)
	CHLA-10	CHLA-10 1411	8.9894E-15	
	CHLA-10	Lapatinib	0.00889308	
**S3D**	CHLA-10 1410	CHLA-10 pLEOC	0.000679651	ANOVA (Bonferroni-Holm post hoc)
	CHLA-10 1411	CHLA-10 pLEOC	0.002019235	
	CHLA-10 1410	CHLA-10 1410 ERBB4	0.000820797	
	CHLA-10 1411	CHLA-10 1410 ERBB4	0.002930856	
	CHLA-10 1410	CHLA-10 1411 ERBB4	0.004832456	
	CHLA-10 1411	CHLA-10 1411 ERBB4	0.017930066	
	CHLA-25 1410	CHLA-25 pLEOC	0.003267103	
	CHLA-25 1411	CHLA-25 pLEOC	0.002007035	
	CHLA-25 1410	CHLA-25 1410 ERBB4	0.049380689	
	CHLA-25 1411	CHLA-25 1410 ERBB4	0.008237516	
	CHLA-25 1410	CHLA-25 1411 ERBB4	0.008404622	
	CHLA-25 1411	CHLA-25 1411 ERBB4	0.007671029	
	SK-N-MC 1410	SK-N-MC pLEOC	0.003055399	
	SK-N-MC 1411	SK-N-MC pLEOC	0.001269884	
	SK-N-MC 1410	SK-N-MC 1410 ERBB4	0.008448919	
	SK-N-MC 1411	SK-N-MC 1410 ERBB4	0.007806959	
	SK-N-MC 1410	SK-N-MC 1411 ERBB4	0.009219662	
	SK-N-MC 1411	SK-N-MC 1411 ERBB4	0.009498295	
**S3E**	TC32 pLEOC	TC32 ERBB4	0.01073	Wilcoxon Matched-Pairs Signed-Ranks
	CHLA-9 pLEOC	CHLA-9 ERBB4	0.00022	
**S4C p-Tyr**	CHLA-9	CHLA-10	0.004360526	ANOVA (Bonferroni-Holm post hoc)
	CHLA-9 ERBB4	CHLA-10	0.02409015	
	CHLA-10	CHLA-10 1410	0.026557832	
	CHLA-10	CHLA-10 1411	0.045368103	
**S4C p-Tyr397**	CHLA-10	CHLA-10 1410	0.019249314	ANOVA (Bonferroni-Holm post hoc)
	CHLA-10	CHLA-10 1411	0.010883253	

SUPPORTING MATERIALS AND METHODS

Expression profiling and data analysis

For gene expression profiling, RNA was extracted from ES cell lines and processed according to manufacturer's instructions (Affymetrix, Inc, Santa Clara, CA). Samples were processed at the Genomics Core at Children's Hospital Los Angeles (CHLA) using the GeneChip Scanner 3000 7G System. Samples were hybridized onto Human Exon 1.0 ST (HuEx) Arrays and processed as required. For DNA analysis arrays, DNA was extracted from cell lines and processed according to manufacturer's instructions (Affymetrix). Each sample was hybridized onto a Genome-Wide Human SNP Array 6.0 (SNP 6.0). For HuEx arrays, CEL files were analysed using Genomics Suite 6.6beta (Partek, Inc, St. Louis, MO). Signal intensities for probe selection regions (PSRs) were quantile normalized by robust multichip averaging (RMA) using na32 annotations. Gene level analyses employed summarizing PSR expression. For SNP 6.0 arrays, CEL files were first analysed using Genotyping Console 4.1.1 (Affymetrix). A quality control threshold for Contrast QC > 0.35 was employed. For subsequent copy-number analysis, SNP 6.0 CEL files were uploaded into Genomics Suite 6.6beta (Partek). Analyses were performed at the Center for Personalized Medicine at CHLA.

RNA isolation, cDNA synthesis and TaqMan real-time quantitative PCR

Total RNA was isolated from CHLA-9, TC32, TC135, TC71, CHLA-10, CHLA-25, CHLA-258 and SK-N-MC ES cells growth as M or S, using RNeasy Total RNA Kit (QIAGEN). Real-time quantitative PCR (qPCR) analysis of cDNA samples was performed using Taqman custom ERBB4 primers and probes (FAM labeled; Applied Biosystems Europe BV, UK) as previously described (Junttila et al, 2003) on a Mastercycler ® ep realplex (Eppendorf). ERBB4 fold changes were calculated by 2^{-ddCT} methods, and Actin was used as an internal control for normalization.

Immunoprecipitation and immunoblotting experiments

ERBB4 tyrosine phosphorylation was evaluated in CHLA-9 and CHLA-10 cells grown as M or S cultures. Cells were pre-incubated for 6 h in medium containing 0.5% FBS, and subsequently treated with 100 ng/ml NRG-1, NRG-2 or HB-EGF ligands for 30 min. Cells were lysed and immunoprecipitation was performed using an anti-ERBB4 Ab (Cell Signaling) as previously described (Kang et al, 2007). Samples were immunoblotted with anti-phosphotyrosine (p-Tyr) and ERBB4 antibodies (Santa Cruz Biotechnology). Total lysates used for ERBB4 immunoprecipitation were probed with anti-Actin Ab (Sigma) as a loading control. ERBB4 tyrosine kinase phosphorylation was additionally assessed in CHLA-10 cells grown as M or S, and treated with 1 μM Lapatinib for 24 h. Immunoblotting was performed as described (Kang et al, 2007). FAK tyrosine phosphorylation (p-Tyr) was evaluated in CHLA-9, CHLA-9-ERBB4, CHLA-10, and 1410 and 1411 ERBB4shRNA CHLA-10 cells, plated as M or S cultures. FAK p-Tyr was also measured in implantation site tumours isolated from mice implanted with CHLA-10 control or CHLA-10 ERBB4 kd cells (three mice per condition). Lysates were immunoprecipitated using an anti-FAK Ab (Santa Cruz), and samples were immunoblotted with anti-p-Tyr, p-FAK Tyr397, or total FAK Abs (Santa Cruz Biotechnology). CHLA-10 cells were plated at semiconfluence, and 24 h later were incubated for 16 h in the presence of 5 μM LY-294002 PI3K inhibitor, 10 μM UO-126 ERK1/2 inhibitor, 5 μM FAK inhibitor 14 or 1 μM FAK inhibitor II. Moreover, ES cells grown as M, S, or under serum-starvation (SS) conditions for 24 h were harvested, lysed, and processed as previously described (Kang et al, 2007). Western blotting was performed with Abs directed to ERBB4, p-Akt Ser 473 and Thr 308; total Akt; p-ERK1/2 and total ERK1/2; and Actin, depending on the experimental setting. Detailed description, origin and dilutions of all antibodies used in this study have been given in Supporting Information **Table S5**.

Human RTK arrays

CHLA-10 cells and ERBB4shRNA-transduced CHLA-10 cells grown as M or S were harvested, washed, and lysed. 1 mg protein lysates were incubated overnight with Phospho-RTK Arrays (R&D Systems) according to the manufacturer's instructions. Bound phospho-RTKs were detected with a pan antiphosphotyrosine antibody conjugated to HRP using *LumiGLO Reserve Chemiluminescent* Substrate (KPL).

Reverse transcription-PCR (RT-PCR) analyses of ERBB4 ligands expressed in ES cells

Total RNA from CHLA-9 and CHLA-10 cells growth as M or S for 24 h, was isolated using RNeasy Total RNA Kit (QIAGEN). Reverse transcription reactions were performed using SuperScript™ II Reverse Transcriptase (Invitrogen) and the PCR was carried out, with little modifications, according to (Adam et al, 1999), using neuregulin-1 and 2 (NRG-1 and NRG-2) primers as described (Revillion et al, 2008), and heparin-binding EGF-like growth factor (HB-EGF), amphiregulin (ARG), betacellulin (BTC), and GAPDH primers as described (Adam et al, 1999), all from Invitrogen.

Wound healing migration assays

The IncuCyte™ live-cell imaging system was used to study cell migration in scratch wound-healing assays. Confluent CHLA-9 and CHLA-10 cells transduced with Scrambled or 1410 and 1411 ERBB4 shRNA constructs were wound-scratched and imaged over a 30 h period at 2 h interval. CHLA-10 cells pretreated with 1 μM Lapatinib were also analysed. The velocity of migration was quantified using Volocity4 analysis software (Improvision/Perkin Elmer) by measuring the distance between the initial and final positions of leading edge cells 30 h after wounding. Experiments were performed six times in duplicate and data was expressed as migration distance vs. time, or as velocity of migration (μm/sec).

ERBB4 rescue experiments and generation of point mutations

ERBB4 silent point mutations were generated using full-length ERBB4 JM-a/CYT-1 and JM-a/CYT-2 sequences and the QuikChange® II site-directed mutagenesis kit (Stratagene), according to the manufacturer's recommendations. To fully eliminate the ERBB4 shRNA knockdown effect, six to seven nucleic acids in the 1410 and 1411 ERBB4 shRNA-targeted regions (in the JM-a/CYT-1 and JM-a/CYT-2 sequences) were mutated. The following primers were used in each case:

Mutated construct	Sense	Antisense
1410 ERBB4 shRNA	ggagaaactgtgaagattccagtagccatcaaa atcctcaatgagacaactggtccc	gggaccagttgtctcattgaggattttgatggctactg gaatcttcacagtttctcc
1411 ERBB4 shRNA	gtccctgaaggaaatcagcgctggcaatattta catcactgacaacagcaacctgtg	cacaggttgctgttgtcagtgatgtaaatattgccagc gctgatttccttcagggac

ERBB4 wild-type constructs (pcDNA3.1 JM-a/CYT-1 and JM-b/CYT-2 isoforms, kind gift of Prof Graham Carpenter) and mutant cDNAs were subcloned into the pENTR vector (Invitrogen) and the final constructs used for rescue or overexpression experiments were obtained by LR reaction with the pLEOC (Gateway-compatible) destination vector, using the Gateway® Technology (Invitrogen). HEK293T cells were then transfected with the JM-a/CYT-1 and JM-a/CYT-2 ERBB4 constructs (either wild type or carrying the silent point mutations) and the pLEOC empty vector, as described above. For ERBB4 overexpression experiments CHLA-9 and TC32 cells were transduced with lentiviral particles containing control pLEOC or wild type JM-a/CYT-1 and JM-a/CYT-2 constructs. CHLA-10, CHLA-25 and SK-N-MC scrambled or ERBB4shRNA cells were transduced with lentiviral particles containing pLEOC and JM-a/CYT-1, JM-a/CYT-2 ERBB4 constructs carrying the silent point mutations. Cells were selected with 10 μg/ml blasticidin (InvivoGen), added every 2 days for one week before being used for experiments.

PI staining/cell cycle experiments

Briefly, CHLA-10 scrambled and ERBB4 kd 1410 and 1411 cells were growth as M and S for 24 h. Cells were then harvested, centrifuged, washed and fixed by addition of ice-cold 95% ethanol and were stored in ethanol overnight at -20°C prior to staining. Cell cycle distribution was determined by staining with 20 μg/ml propidium iodide (PI, Sigma Aldrich) and 100 μg/ml RNase A (Sigma Aldrich). Flow cytometric analysis was performed using a CyAn™ ADP flow cytometer (Beckman Coulter) and data were acquired and analysed using the Summit software (Beckman Coulter). Apoptosis was analysed by flow cytometry and quantified as the sub-G_1 (G_0) population/fraction.

Boyden chamber migration and invasion assays

Invasiveness and migration were quantified using Matrigel BD coated or uncoated Boyden chambers, respectively, according to the manufacturer's instructions (BD Biosciences, Oxford, UK). $5x10^4$ or $1.5x10^5$ CHLA-9, CHLA-10, CHLA-25 and SK-N-MC cells transduced with Scrambled or ERBB4 shRNA constructs (1410 and 1411), and TC32 and CHLA-9 cells transduced with p.LEOC or ERBB4 JM-a/CYT-1 and JM-a/CYT-2 constructs were plated in 250 μl serum-free medium in each insert. Medium containing 10% FBS (Invitrogen) was added in the lower chamber as a chemoattractant. Cells were incubated at 37 °C for 16 or 24 h (migration or invasion, respectively). The inserts were incubated for 20 min in 70% Ethanol and cells were stained using 0.5% (w/v) Crystal violet and 1 mg/ml Hoechst 33342 (Sigma) for 30 min. In some experiments, migration and invasion were evaluated in CHLA-10 cells in the presence of 5 μM LY-294002 PI3K inhibitor, 5 μM FAKinh14, or 1 μM FAKinhII added to the upper and lower chambers. CHLA-10 cells transfected with scrambled (Dharmacon), Rac1 siRNA (FlexiTube siRNA, S102655051, Dharmacon), AKT1 and AKT2 siRNA oligos (M-003000-03-0005 and M-003001-02-0005;

Thermo Scientific), or cells transfected with Addgene Myc-tagged Rac1T17 dominant negative (Nobes & Hall, 1999), or HA-tagged AKT dominant negative (Zhang et al, 2003) constructs were also used in these studies. Migration and invasion rates were quantified by counting the number of cells in five random fields for each condition, and mean values from three independent experiments were used.

Cell spreading assays and quantification

Confluent CHLA-9 CHLA-10 cells transduced with scrambled or 1410 and 1411 ERBB4shRNA constructs were harvested by trypsinizaytion and replated for 2 h on plastic dishes (for Rho GTPase activation experiments) or glass coverslips. Cells on glass were then fixed with 4% paraformaldehyde (PFA) for 15 min and permeabilized with 0.1% Triton X-100. Polymerized actin was stained using TRITC-Phalloidin (Sigma Aldrich), followed by 10 min incubation with 5 μg/ml of the nuclear dye Hoechst 33342 (Sigma Aldrich). Cells were imaged using a 63×, 1.25 NA objective on a Leica SP2 confocal microscope (Leica Microsystems, UK). All acquisition parameters were kept constant throughout each experiment and cell spreading was quantified 2 h after cell plating, according to (Price et al, 1998).

Histological staining and IHC of murine tumour specimens

Animals were humanely sacrificed and the primary tumours and lungs were collected, formalin-fixed and paraffin-embedded following staining with haematoxylin and eosin (H&E) for histological analysis, using a Zeiss Axioplan2 fluorescence microscope (Carl Zeiss GmbH). ERBB4 expression was analysed by IHC and was performed following deparaffinization and antigen retrieval (Ventana CC1), using an anti-ERBB4 antibody (Santa Cruz Biotechnology) diluted 1:50 and Ventana discovery XT system. Detection was carried out using Ultramap anti-Rabbit HRP (Ventana). Sections were counterstained with

hematoxylin (Vector Labs), and images were acquired with a Zeiss Axioplan2 fluorescence microscope (Carl Zeiss GmbH).

WST-1 cell viability assay

Cell viability was measured in CHLA-10 cells plated at 2.5×10^4 cells/well in 96-well plates and treated with 5 μM LY-294002, 10 μM UO-126, 5 μM FAKinh14 or 1 μM FAKinhII; cells were monitored for a further 40 h. In some experiments, semiconfluent cultures of CHLA-10, CHLA-25, and SK-N-MC cells transduced with scrambled or ERBB4 shRNA constructs, and seeded at 2×10^4 cells/well in 96-well plates, were monitored for 24 h. After this time, the WST-1 reagent (Roche) was added and the formation of the formazan dye was allowed to proceed for 4 h at 37°C. WST-1 conversion was calculated as the difference between the values obtained at 440 nm and 620 nm, after subtraction of background values of WST-1 incubated in the absence of cells, measured on a microplate reader (Thermo Scientific Varioskan® Flash). The graphs were generated considering the highest value of absorbance from 3 experiments as 100%. The rest of the values were calculated with reference to this maximum value. Data were expressed as percentage ± SD of at least three independent experiments.

Patient characteristics

Nineteen paired primary and metastatic samples were obtained from ES patients; of them, sixteen were treated within the London Sarcoma Service between 1996 and 2010 and were obtained from the Royal National Orthopaedic Hospital (RNOH) Musculoskeletal Biobank (approved by the Cambridgeshire Research Ethics committee, Cambs. UK; Reference Number: 09/H0304/78). Nine patients had bone marrow metastases, all present on diagnostic staging examination. The other 7 patients had lung metastases that were resected a median of 40 months (range 8-88) after diagnosis. The clinical characteristics of these patients are

summarized in Supplementary Table S2. A further three paired samples were obtained from patients treated at AC Camargo Hospital. In addition, a tissue microarray (TMA) of diagnostic biopsy specimens from 94 ES patients with ES, diagnosed and treated at the AC Camargo Hospital from 1980 to 2008, were available for ERBB4 expression analysis. The median age at diagnosis of ES was 15 years (range 4 months - 52 years). Patients were treated with multi-agent chemotherapy, local control with radiotherapy and surgery, or a combination of these as appropriate for the period of the study and primary tumor site. Demographic data available from 48 patient records were used to examine potential associations between clinico-pathological characteristics and ERBB4 expression (Supplementary Table S3). In addition, outcome data from 51 patients with localized disease at diagnosis were available for disease-free survival analysis.

ERBB4 immunohistochemistry on pathologic specimens

ERBB4 immunohistochemistry staining was carried out using an anti-ERBB4 Ab (C-18, Santa Cruz Biotechnology) on a Leica Bond Bond-Max automated IHC Stainer. Semi-quantitative scoring of histological staining was performed using a linear scoring system*:* 0-absent staining*;* 1-staining in 0-25%; 2-staining in 26-50%; and 3-staining in 51-75% of the analysed tissue and interpreted by two independent pathologists who were blinded to the clinical information. Positive control tissues included carcinoma positives for ERBB4. As a negative control, the ERBB4 antibody (Ab) was omitted and substituted by an isotype control (rabbit IgG; Santa Cruz Biotechnology). Immunostaining with anti-ERBB4 Ab was performed on 5 μm patient sections from the TMA and applied in accordance with the manufacturer's instructions (Santa Cruz Biotechnology). Slides were first incubated with a peroxidase-blocking reagent for 5 minutes and then with primary anti-ERBB4 Ab for 30 minutes followed by a visualization step using DAB staining. After a final wash, the slides were counterstained with hematoxylin. Scoring was performed according to the pathologist

criteria from AC Camargo group, who followed the quantification of the average staining intensity as 0- negative, 1- weak, 2- moderate, or 3- strong.

SUPPORTING REFERENCES

1. Adam RM, Borer JG, Williams J, Eastham JA, Loughlin KR, Freeman MR (1999) Amphiregulin is coordinately expressed with heparin-binding epidermal growth factor-like growth factor in the interstitial smooth muscle of the human prostate. Endocrinology 140**:** 5866-5875

2. Junttila TT, Laato M, Vahlberg T, Soderstrom KO, Visakorpi T, Isola J, Elenius K (2003) Identification of patients with transitional cell carcinoma of the bladder overexpressing ErbB2, ErbB3, or specific ErbB4 isoforms: real-time reverse transcription-PCR analysis in estimation of ErbB receptor status from cancer patients. Clin Cancer Res 9**:** 5346-5357

3. Kang HG, Jenabi JM, Zhang J, Keshelava N, Shimada H, May WA, Ng T, Reynolds CP, Triche TJ, Sorensen PH (2007) E-cadherin cell-cell adhesion in ewing tumor cells mediates suppression of anoikis through activation of the ErbB4 tyrosine kinase. Cancer research 67**:** 3094-3105

4. Nobes CD, Hall A (1999) Rho GTPases control polarity, protrusion, and adhesion during cell movement. The Journal of cell biology 144**:** 1235-1244

5. Price LS, Leng J, Schwartz MA, Bokoch GM (1998) Activation of Rac and Cdc42 by integrins mediates cell spreading. Molecular biology of the cell 9**:** 1863-1871

6. Revillion F, Lhotellier V, Hornez L, Bonneterre J, Peyrat JP (2008) ErbB/HER ligands in human breast cancer, and relationships with their receptors, the bio-pathological features and prognosis. Annals of oncology : official journal of the European Society for Medical Oncology / ESMO 19**:** 73-80

7. Zhang HM, Yuan J, Cheung P, Luo H, Yanagawa B, Chau D, Stephan-Tozy N, Wong BW, Zhang J, Wilson JE et al (2003) Overexpression of interferon-gamma-inducible GTPase inhibits coxsackievirus B3-induced apoptosis through the activation of the phosphatidylinositol 3-kinase/Akt pathway and inhibition of viral replication. The Journal of biological chemistry 278**:** 33011-33019
